# Individual or successiveseed priming with nitric oxide and calcium toward enhancing salt tolerance of wheat crop through early ROS detoxification and activation of antioxidant defense

**DOI:** 10.1186/s12870-024-05390-0

**Published:** 2024-07-31

**Authors:** Rasha M. El-Shazoly, H. M. A. Hamed, Mahmoud M. El-Sayed

**Affiliations:** 1 Botany and Microbiology Department Faculty of Sciences, New Valley Univ, Al-Kharja, New Valley, 72511 Egypt; 2 Soils and Water Science Department, Faculty of Agriculture, Al-Azhar Univ, Assiut, Egypt

**Keywords:** Irrigation water applied, Irrigation water productivity, Nitric oxide, Stress alleviation, Seed priming, Water consumptive use

## Abstract

Despite the considerable efforts reported so far to enhance seed priming, novel ideas are still needed to be suggested to this sustainable sector of agri-seed industry. This could be the first study addressing the effect of nitric oxide (NO) under open field conditions. The impacts of seed redox-priming using sodium nitroprusside (SNP) and osmo-priming with calcium chloride (CaCl_2_), both applied individually or successively, were investigated under salinity stress conditions on wheat plants (*Triticum aestivum L.*). Various parameters, including water relations, growth, yield, photosynthetic pigments, and antioxidant activities (enzymatic and non-enzymatic), were recorded to assess the outcomes of these priming agents on mitigating the negative impacts of salinity stress on wheat plants. Water consumptive use (ETa) and irrigation water applied (IWA) decreased with seeds priming. Successive priming with SNP + CaCl_2_ induced the greatest values of crop water productivity (CWP), irrigation water productivity (IWP), seed index, grain yield and grain nitrogen content.Under salinity stress, the dry weight of plants was decreased. However, hydro-priming and successive chemical priming agents using combinations of calcium chloride and sodium nitroprusside (CaCl_2_ + SNP & SNP + CaCl_2_) preserved growth under salinity stress.Individual priming with sodium nitroprusside (SNP) and calcium chloride (CaCl_2_) resulted in the lowest recorded content of sodium in the shoot, with a value of 2 ppm. On the other hand, successive priming using CaCl_2_ + SNP or SNP + CaCl_2_ induced the contents of potassium in the shoot, with values of 40 ppm and 39 ppm, respectively. Malondialdehyde decreased in shoot significantly withapplicationof priming agents. Successive priming with CaCl_2_ + SNP induced the highest proline contents in shoot (6 µg/ g FW). The highest value of phenolics and total antioxidants contents in shoot were recorded under successive priming using CaCl_2_ + SNP and SNP + CaCl_2_.Priming agents improved the activities of ascorbate peroxidase and catalase enzymes. The successive priming improved water relations (ETa, IWA, CWP and IWP) and wheat growth and productivity under salinity stress more than individual priming treatments.

## Introduction

Recently, climate change, the eco-toxicological practices, and environmental stressors have posed many threats to agriculture sustainability all over the globe. They have generated serious challenges to biodiversity, food security, and sustainable agriculture resources [1, 2]. Recent global monitoring of climate changes has revealed a serious disturbing trend of buildup of extreme weather consequences, including the appearance of more severe and frequent periods of drought. Salt stress affects more than 20% of cultivated land worldwide as a consequence of the growing use of poor-quality water for irrigation and hence soil salinization [3]. The recorded increase in the concentration of atmospheric greenhouse gases, particularly CO_2_, and climate warming is unequivocal [4]. While elevated CO_2_ levels may offer benefits for plants, they indirectly pose threats of heat stress, drought, and salinity [5]. The challenges projected from future climate change and the resulting impacts on global sustainable agriculture propose negative impacts on crop yields averages and yield variability [5]. On a global scale, it is obvious that among all abiotic stressors, salinity and drought are the main limiting factors of both growth and productivity of crops [6]. The most sensitive stages for crop development affected by abiotic stresses are seed sprouting and seedling establishment. Disturbance in plants water absorption occur as a result of hyperosmotic stress and salinity [7]. Seed germination and seedling sprouting time are negatively hindered due to high salt concentration, delayed germination is associated with high salt concentrations, and this correlation is also influenced by whether the salt concentration is low or high. Low salt concentration (i.e., below the optimum level) induces dormancy, whereas high salt concentrations above the optimum level reduce the percentage of germination and hamper the process of sprouting due to water loss as a consequence of increased transpiration and high salt accumulation around the plant roots [8]. Many molecular and physiological mechanisms are adapted by the plants to manage the stress condition such as ionic tolerance, osmotic tolerance, and tissue tolerance [9, 10]. Kranner et al. [11] characterized the applications of stress in seed biology. The authors discussed the seed life cycle in light of the eustress-distress concept. In the field of seed and plant science, the concept of stress has been adopted from biomedical sciences and is divided into two types. The first type, known as eustress, activates a positive effect and stimulates a response. The second type, distress, causes a negative effect. According to this classification, seed priming (as an artificial process) could be considered eustress rather than distress as priming application shows similarity with the natural process of hydration-rehydration cycles, which seeds undergo after sowing in the soil [12]. Sustaining crop productivity and the ability to adapt to frequent stress impacts is a top urgent issue [13]. Thus, the priming technique is a very promising tactic used to enhance plant stress tolerance [14]. In brief, priming refers to the pre-exposure of seeds to stimulating factors, which helps plants develop tolerance towards future abiotic or biotic stressors [15]. It is considered as a potential technique to enhance stress tolerance, and it is connected to alert mode or “plant stress memory” [15]. As it is considered a cost-efficient approach [16], it is recently a prominent strategy for climate change scenarios [17]. There are many types of priming techniques and their mechanisms; hydro-priming, Osmo priming, Chemo-priming, and Redox priming [18–20]. Redox priming is a technique that utilizes natural and/or synthetic redox compounds, including antioxidants such as ascorbic acid, glutathione, and tocopherol, as well as hydrogen peroxide and sodium nitroprusside (SNP). This approach has been proved to improve seed germination and seedling establishment, both under stressed and unstressed conditions [21, 22]. Exogenous application of gaseous nitric oxide (NO) in the form of SNP has gained importance. Nitric oxide has volatile and lipophilic nature and acts as a free radical [23]. Besides its regulatory roles in plants in improving seed germination and seedling growth [24], it also plays a protective role against different abiotic stresses particularly salinity [25]. Earlier studies have suggested its role in improving salt tolerance in many plants such as tomato, rice and wheat [26–28].

In plants, if large amount of calcium applied in the field it could promote calcification of soil, particularly, in the alkali saline soil [29]. Therefore, CaCl_2_ as seed priming agent can be used as a sustainable agriculture and environmentally friendly tool to enhance crop tolerance. Moreover, seed priming with CaCl_2_ offers prominent economic advantages when compared to within-crop spray treatments, as it can easily be applied by growers or seed distributors. The application of calcium as priming agent can increase the concentration of Ca^2+^ in plants, particularly upon activation of resistance [30]. The effect of seed osmopriming (with CaCl_2_) led to establishment of early tolerance mechanisms on wheat plant, which resulted in increased yield and crop allometry and improved leaf area index, crop growth rate and productivity under drought stress [31]. Wang et al. [32] found that pretreatment of seeds with CaCl_2_ enhanced tolerance to salt and cold stress. The results obtained from this study indicate the role of CaCl_2_ as priming agent in activating resistance mechanisms in rice seedlings. Hence, developing a cost effective and economically feasible technique to overcome salt or drought stresses is a challenge [33, 34]. Globally, among various studies that have been carried out to deal with the osmotic stress, seed priming is a very promising strategy possessing the ability to improve crops yields and yield quality through alleviating salinity and drought stresses unfavorable outcomes.

Wheat is very important cereal crops; it is the main source of carbohydrates and a major staple food around the globe [35, 36]. Under unfavorable conditions of water deficit and salinity stresses, wheat seed germination and seedling establishment also experience the aforementioned negative impacts on physiological and biochemical attributes and vast metabolic processes [37]. Subsequently, it seriously reduces the percentage of germination, growth, biomass production and grain yield in wheat plant [38]. In the human diet, wheat is considered a source of over 20% of calories [39]. Globally, it covers about a fifth of the total cereals-cultivated land [40]. As the demand for cereals is expected to expand by 2050 to reach about 3 billion tonnes, wheat cultivation has increased [41]. However, as a consequence of climate change, it is expected that its global production may decrease by about 1.9% in the second half of this century, and the negative impact of climate change will be more obvious in Southern Asian and African countries, with a predicted decline in yield of about16% and 15%, respectively, by 2050 [42]. Similarly, it is predicted that per every degree Celsius rise in temperature, there will be a reduction in wheat production by about 6% globally [43]. The continued scenarios of climate change, particularly salinity and drought, are predominant factors challenging the global wheat production.

Considering the aforesaid factors, working on anti-salinity and anti-drought techniques and strategies confronting the growing abiotic stress projected from climatic change is crucial to achieving sustainable food security. The present study hypnotized that different seed priming techniques (hydro, redox and chemical) and their application individually or successively can provoke regulated priming memory permanent till plant maturity, a much-expected technique for achieving sustainable agriculture under the anticipated scenario of climate change.

This study examined the effect of different priming methods (individually or successively) on wheat water relations, yield, wheat photosynthetic pigments, enzymatic and non-enzymatic antioxidant activities, and their related traits under changing scenarios of the climate. This study could be the first study addressing the effect of NO under open field conditions. 

The following questions were addressed: 1) Do priming agents improve plant growth and production performance beyond the germination stage under field conditions? 2) Do priming agents improve salinity tolerance and yield production? 4) which priming agent(s) can improve the salinity tolerance of the test plant? 5) Are the beneficial effects resulting from chemical priming parallel to those from redox priming or hydro-priming? 6) Are the beneficial effects resulting from individual chemical priming or redox priming parallel to those from successive priming?

## Materials and methods

### The experimental site

A field experiment was carried out at a privet farm in Eneiybes, Juhayna, Sohag Governorate, Egypt, which is located at 26° 73- 67^=^ N latitude and 31° 47- 56^=^ E longitude during two consecutive growing winter seasons of 2019/20 and 2020/21 to assess the effect of seed priming on wheat yield and water productivity under salinity stress.

### Climate conditions

The climate condition of the studied area represented the Sohag Governorate (Upper Egypt). Monthly average agro-meteorological data at the experimental site and reference evapo-transpiration (ETo) values for the two seasons were obtained from the meteorological station in Sohag, Egypt, and are presented in Table 1.

**Table 1:** Monthly average meteorological data values of the studied area

Year	Months	Temperature (^o^C)			WS (m sec^−1^)	SR (MJ/m^2^/day)	RH (%)	ET_o_ (mm)
		Max	Min	Ave				
	Dec	21.0	7.5	14.3	2.5	15.0	61.5	2.7
	Jan	18.6	4.7	11.6	2.3	15.0	59.0	2.4
	Feb	21.5	6.9	14.2	2.6	18.5	50.0	3.1
	Mar	26.2	9.9	18.0	3.0	22.5	40.0	4.6
	Apr	30.1	13.9	22.0	3.3	24.5	35.5	5.9
	May	37.2	20.3	28.8	3.2	27.0	33.0	7.3

Source: Meteorology Station of Sohag, Egypt

*WS* Wind speed, *SR* Solar radiation, *RH* Relative humadity, *ETo* Evapotranspiration

### Soil analysis of the experimental site

Soil samples were taken from two close sites representing normal soil (unsaline soil) and nearby saline soil with a 15- cm increment and down to 60-cm soil depth using a spiral auger. In the laboratory, the collected samples were air- dried, ground, and sieved (particle size < 2 mm). The prepared samples were subjected to chemical and physical analysis according to Klute [44] and Page et al. [45]. The data for soil analysis were presented in Tables 2 and 3. Also, undisturbed soil samples were taken using the core method technique.

**Table 2:** Some soil properties of the experimental site (unsaline soil)

Property	Soil depth (cm)			
	**0–15**	**15–30**	**30–45**	**45–60**
**pH (1: 2.5)**	**7.77**	**7.80**	**7.87**	**8.00**
**EC**_**e**_** (dS m**^**−1**^**)**	**0.85**	**0.87**	**0.90**	**0.91**
**CaCO**_**3**_** (%)**	**5.25**	**5.28**	**5.56**	**5.71**
**OM (%)**	**2.12**	**1.87**	**1.45**	**1.13**
**Available N (ppm)**	**73.34**	**70.22**	**67.45**	**62.86**
**Available P (ppm)**	**12.13**	**11.37**	**10.75**	**10.26**
**Available K (ppm)**	**255.24**	**248.78**	**236.54**	**232.67**
**Sand (%)**	**51.00**	**50.80**	**50.50**	**50.20**
**Silt (%)**	**30.00**	**30.00**	**30.00**	**30.00**
**Clay (%)**	**19.00**	**19.20**	**19.50**	**19.80**
**Texture class**	**loam**	**loam**	**loam**	**loam**
**Bd (Mg m**^**−3**^**)**	**1.38**	**1.40**	**1.41**	**1.43**
**SP (%)**	**66.00**	**66.32**	**66.67**	**67.00**
**FC (%)**	**35.70**	**35.34**	**35.60**	**35.83**
**WP (%)**	**17.65**	**17.50**	**17.50**	**17.34**
**AW (%)**	**18.05**	**17.84**	**18.10**	**18.49**
**HC (m day**^**−1**^**)**	**0.65**			

*OM* Organic matter, *Bd* Bulk desity, *SP* Saturation percent; *FC* Field capacity, *WP* Wilting point, *AW* Available water, *HC* Hydraulic conductivity

**Table 3:** Some soil properties of the experimental site (Saline soil)

Property	Soil depth (cm)			
	**0–15**	**15–30**	**30–45**	**45–60**
**pH (1: 2.5)**	**7.98**	**8.06**	**8.15**	**8.20**
**EC**_**e**_** (dS m**^**−1**^**)**	**7.15**	**6.57**	**6.25**	**6.24**
**CaCO**_**3**_** (%)**	**5.32**	**5.37**	**5.74**	**5.95**
**OM (%)**	**2.00**	**1. 70**	**1.32**	**1.07**
**Available N (ppm)**	**74.24**	**69.95**	**66.78**	**63.12**
**Available P (ppm)**	**12.17**	**11.34**	**10.64**	**10.42**
**Available K (ppm)**	**260.14**	**252.67**	**234.68**	**230.00**
**Sand (%)**	**50.00**	**51.00**	**50.40**	**50.20**
**Silt (%)**	**30.00**	**30.00**	**29.80**	**29.70**
**Clay (%)**	**20.00**	**19.00**	**19.80**	**20.10**
**Texture class**	**loam**	**loam**	**loam**	**loam**
**Bd (Mg m**^**−3**^**)**	**1.36**	**1.41**	**1.43**	**1.44**
**SP (%)**	**67.00**	**67.12**	**67.45**	**67.65**
**FC (%)**	**34.95**	**34.67**	**35.20**	**35.64**
**WP (%)**	**18.34**	**18.00**	**18.75**	**18.56**
**AW (%)**	**16.61**	**16.67**	**16.45**	**17.08**
**HC (m day**^**−1**^**)**	**0.62**			

*OM* Organic matter, *Bd* Bulk desity, *SP* Saturation percent; *FC* Field capacity, *WP* Wilting point, *AW* Available water, *HC* Hydraulic conductivity

### Experimental design: salinity stress

The tested salinity stress treatments (Seven treatments), with three replicates, were arranged in a completely randomized design. The field experiment was conducted using treatment “no priming” (under unsaline soil) as control and six treatments under saline soil conditions as follows: no priming, hydro-priming, individual osmo-priming calcium chloride CaCl_2_, individual redox-priming sodium nitroprsside SNP, successive priming CaCl_2_ + SNP, and successive priming SNP + CaCl_2_.

### Growth conditions and treatments

Well-selected grains of wheat (*Triticum aestivum L*.) were rinsed thoroughly in distilled water and then soaked in the priming agents Hydro H_2_O, CaCl_2_ (15 mM), and SNP (0.5 mM). The concentrations of CaCl_2_ and SNP applied in this experiment were selected according to preliminary experiments (data not shown).Grains were soaked for 12 h, then air-dried and sowed in the soil in the case of individual priming or re-soaked in an alternate successive priming agent for additional 12 h in the case of successive priming.

### Agronomic practices

All the agriculture practices were carried out according to the given recommendations by the Egyptian Ministry of Agriculture and applied as commonly used for wheat plantations. Wheat plants were harvest after 160 days of planting. Ammonium nitrate (33.5% N) was used as nitrogen fertilizer and applied in two equal doses at a level of 240 kg ha^−1^. The first dose was applied before post-planting irrigation, and the second one was applied before the second irrigation, particularly at the stage of tillering. Calcium superphosphate was used as phosphorus fertilizer (15.5% P_2_O_5_). It was added to the soil at a level of 200 kg ha^−1^. A single dose was added during the preparation of the soil. potassium sulphate was used as the source of potassium fertilizer (48% K_2_O). It was applied to the soil in two equal doses at a level of 100 kg ha^−1^, concurrent with the addition of nitrogen fertilizer.

### Water consumptive use (ETa) and irrigation water applied (IWA)

Actual evapotranspiration of the wheat crop was estimated by the soil sampling method to calculate soil moisture according to the method of Israelsen and Hansen [46] using the following formula:

CU = (θ2—θ1) Bd * ERZ where CU is the amount of consumptive water use (mm),

θ2 is the soil moisture percentage after irrigation, θ1 is soil moisture percentage before the following irrigation, Bd is bulk density (g. Cm^−3^), and ERZ is the effective root zone.

The experimental plots of 60 cm soil depth received an amount of water to boost the moisture up to field capacity. The irrigation water applied (IWA) in each irrigation treatment was calculated to be equal to the difference between moisture at the field capacity and the soil moisture content before irrigation.

### Irrigation water productivity and crop water productivity

The Irrigation water use efficiency (IWUE) was calculated according to Du et al. [47] using the following equation: IWP (kg/ m^3^) = Y/ I, Where Y is the grain yield (kg ha^−1^) and I is the irrigation water applied (m^3^ ha^−1^).

Crop water productivity (CWP) describes the efficiency of the water applied for yield production. It was calculated, as described by Zwart and Bastianssen [48] as follows:

CWP (kg m^−3^) = Y/ Eta [ETa is the seasonal actual water consumptive use (m^3^ ha^−1^)].

### Determination of photosynthetic pigments

Chlorophylls and carotenoids concentrations were conducted using equations as cited by Lichtenthaler [49]. To extract pigments, fresh leaf samples were suspended in 10 ml of 95% ethyl alcohol at 60ºC, until colorless. Absorbance readings were determined spectrophotometercaly.

#### Preparation of plant extract

Fresh plant samples were extracted according to Padmaja et al. [50]. The resultant supernatant was used for determination of antioxidant enzymes (catalase and peroxidase), non-enzymatic antioxidants (free phenolic and total antioxidant [DPPH]), and metabolites (soluble proteins). While proline and MDA are determined in shoots only and have their own extraction method.

## Shoot stress markers

### Determination of membrane damage

To assess the membrane damage in shoot samples, lipid peroxidation (MDA) was conducted according to Hodges et al. [51]. The results were expressed as μM MDA g^−1^ FW.

#### Determination of proline

Free proline was extracted and measured as reported by Bates et al. [52]. Proline concentration was expressed as mg proline g^−1^ FW.

#### Total antioxidant activity (DPPH) and free phenolics

DPPH-stable free radical scavenging activity was determined by the method of Blois [53]. The inhibition percentage (I) was calculated as radical scavenging activity as follows I = (Abs control-Abs sample)/Abs control X 100.

The determination of Phenolics was conducted according to Kofalvi and Nassuth [54], and its concentration was expressed as µg g^−1^ FW.

#### Catalase (EC 1.11.1.6)

Catalase (CAT) activity was conducted by following the method of Aebi, (1984) [55].

#### Peroxidase (EC 1.11.1.7)

Peroxidase (POD) activity was determinedfollowing the method described by Tatiana et al. [56].

#### Assay of metabolites: soluble proteins

Protein contents in the shoot samples were measuredas described by Lowry et al. [57].

#### Ionic analysis

The plant material extractions were conducted by the mixed acid digestion procedure, as reported by Allen [58]. The determination of cations (Na^+^ and K^+^) assessed using Carl Zeiss flame photometer due to the high sensitivity of the flame emission method for cations [59].

#### Crop measurements

At the end of experimental time (harvest stage), ten random plants were chosen from a square meter from each treatment in order to estimate the following parameters: grain yield, seed index (weight as g/1000 grains), straw yield, and nitrogen percentage in grain. The estimation of grain and straw yield was assessed by collecting data from the centric area of each treatment. Four square meters (2 m x 2 m) were used, and the data were converted to yield/ ha.

#### Statistical analysis

The data was collected in three replicates from six measurements from two independent experiments. The Analysis of variance (ANOVA) was conducted using the SPSS statistical 11.0 package. The comparison of the means for significant differences was performed using Duncan’s multiple range tests at p ≤ 0.05 as a posthoc test. All the assessed attributes were analyzed with Principal Component Analysis (PCA) variance regression ordination. The heatmap and scatter plot were generated using ggplot packages and visualization of corrplot, integrated into the R software (RStudio). The data (mean values) was normalized into a standard range of ± 1, in order to perform the analysis.

## Results 

In the present investigation, an attempt was done to explore the effect of seed priming with nitric oxide or calcium chloride at different methods (individually or successively) on the performance of wheat grain germination, early seedling establishment, and crop production when germination occurs under salt stress under open field conditions. This study could be the first study addressing the effect of NO under field conditions. This work designed to further and deeper understand how primed seeds effectively take advantage from nitric oxide and calcium to downstream subsequent defense, the present investigation evaluated the events of oxidative stress, with focus on stress markers and antioxidant systems that may be activated after the exposure to salt stress. 

### Hydraulic conductivity (HC) and bulk density (Bd) of soil

Hydraulic conductivity as affected by salinity and seeds priming in the first season of 2019/20 and the second season 2020/21 is represented in Table 4. The hydraulic conductivity was significantly increased due to the seeds priming.

**Table 4:** Effect of Salinity and seeds priming on hydraulic conductivity (HC) and bulk density (Bd) of soil

priming	HC (m day^−1^)		mean	RC	Bd (Mg m^−3^)		mean	RC
	**2019/20**	**2020/21**			**2019/20**	**2020/21**		
**No priming (unsaline soil)**	**0.62 ± 0.06e**	**0.68 ± 0.09d**	**0.65**	**0.00**	**1.42 ± 0.05a**	**1.43 ± 0.07a**	**1.43**	**0.00**
**No priming (saline soil)**	**0.61 ± 0.07e**	**0.63 ± 0.05e**	**0.62**	**1.52**	**1.38 ± 0.04b**	**1.37 ± 0.06b**	**1.38**	**-4.01**
**Hydro** **(H**_**2**_**O)**	**0.65 ± 0.04d**	**0.67 ± 0.06d**	**0.66**	**28.18**	**1.37 ± 0.03b**	**1.37 ± 0.05b**	**1.37**	**-8.37**
**CaCl**_**2**_	**0.91 ± 0.05b**	**0.90 ± 0.03b**	**0.91**	**28.18**	**1.31 ± 0.06c**	**1.32 ± 0.08c**	**1.32**	**-8.37**
**SNP**	**0.86 ± 0.06c**	**0.86 ± 0.05c**	**0.86**	**24.42**	**1.30 ± 0.02c**	**1.29 ± 0.03c**	**1.30**	**-10.04**
**CaCl2 + SNP**	**0.95 ± 0.08a**	**0.94 ± 0.06a**	**0.95**	**31.22**	**1.25 ± 0.07d**	**1.24 ± 0.05d**	**1.25**	**-14.46**
**SNP + CaCl2**	**0.96 ± 0.07a**	**0.95 ± 0.08a**	**0.96**	**31.94**	**1.23 ± 0.04d**	**1.23 ± 0.06d**	**1.23**	**-15.85**

*RC* relative change, *HC* hydraulic conductivity, *Bd* Bulk desity

The value hydraulic conductivity reached its peak (0.96 m day^−1^) with SNP + CaCl_2_ during 1st season. The lowest value of hydraulic conductivity (0.61 m day^−1^) was observed with no priming in the 1st season. On the basis of average from both growing seasons, values of hydraulic conductivity were 0.65, 0.62, 0.66, 0.91, 0.86, 0.95 and 0.96 m day^−1^ at control, no priming, hydro, and CaCl_2_, SNP, CaCl_2_ + SNP and SNP + CaCl_2_, respectively. The seed priming, regarding their effect on hydraulic conductivity, could be arranged descendingly in the following order: SNP + CaCl_2_ > CaCl_2_ + SNP > CaCl_2_ > SNP > hydro > control > no priming for both seasons.

Bulk density as affected by salinity and seeds priming in the first season of 2019/20 and the second season of 2020/21 is represented in Table 4. The bulk density was significantly increased due to the seeds priming. The bulk density value reached its peak (1.43 Mg m^−3^) with control during 2nd season. The lowest value of bulk density (1.23 Mg m^−3^) was observed with SNP + CaCl_2_ in the 1st and 2nd season. On the basis of average from both growing seasons, values of bulk density were 1.43, 1.38, 1.37, 1.32, 1.30, 1.25, and 1.23 Mg m^−3^ at control, no priming, hydro and CaCl_2_, SNP, CaCl_2_ + SNP and SNP + CaCl_2_, respectively. The seeds priming, regarding their effect on bulk density, could be arranged descendingly in the following order of: control > No > hydro > CaCl_2_ > SNP > CaCl_2_ + SNP > SNP + CaCl_2_ for both seasons.

### Wheat irrigation water applied (IWA) and water consumptive use (ETa)

Salinity greatly reduces the production of wheat crop in arid and semi-arid regions. The data of water consumptive use (ETa) and irrigation water applied (IWA) are shown in Table 5 for wheat that was affected by seeds priming and salinity during the two-growing season (2019/20 and 2020/21). Generally, the amount of water consumptive use (ETa) and irrigation water applied (IWA) decreased with seeds priming. The calculated amounts of applied irrigation water (IWA), on the basis of the average of two growing seasons, were 6476.45, 6493.53, 6487.01, 6464.48, 6436.63, 6423.14 and 6413.78 m3 ha^−1^ while water consumptive use (ETa) was 4859.65,4848.46, 4821.77, 4811.04, 4796.05, 4785.29 and 4792.71 m^3^ ha^−1^ at control,no priming, hydro and CaCl_2_, SNP, CaCl_2_ + SNP, and SNP + CaCl_2_, respectively.

**Table 5:** Effect of Salinity and seeds priming on wheat water consumptive use (ETa) and irrigation water applied (IWA)

priming	ETa (m^3^ ha^−1^)		mean	RC	IWA (m^3^ ha^−1^)		mean	RC
	**2019/20**	**2020/21**			**2019/20**	**2020/21**		
**No priming (unsaline soil)**	**4843.96 ± 2.50a**	**4875.35 ± 6.90a**	**4859.65**	**0.00**	**6464.25 ± 8.12c**	**6488.65 ± 6.21b**	**6476.45**	**0.00**
**No priming (saline soil)**	**4838.18 ± 12.0b**	**4858.73 ± 10.0b**	**4848.46**	**-0.79**	**6483.50 ± 2.74a**	**6503.56 ± 2.94a**	**6493.53**	**0.16**
**Hydro** **(H**_**2**_**O)**	**4809.07 ± 3.92c**	**4834.47 ± 3.14c**	**4821.77**	**-1.01**	**6476.91 ± 11. 4b**	**6497.12 ± 7.2b**	**6487.01**	**-0.19**
**CaCl**_**2**_	**4794.84 ± 4.18d**	**4827.24 ± 829c**	**4811.04**	**-1.01**	**6449.27 ± 11.66d**	**6479.70 ± 11.52c**	**6464.48**	**-0.19**
**SNP**	**4784.79 ± 3.34e**	**4807.30 ± 5.6d**	**4796.05**	**-1.33**	**6421.23 ± 3.83e**	**6452.02 ± 8.14d**	**6436.63**	**-0.62**
**CaCl2 + SNP**	**4776.76 ± 2.86e**	**4793.83 ± 3.45e**	**4785.29**	**-1.55**	**6413.83 ± 6.63e**	**6432.45 ± 14.31e**	**6423.14**	**-0.83**
**SNP + CaCl2**	**4783.62 ± 5.04e**	**4801.80 ± 4.35d**	**4792.71**	**-1.40**	**6397.96 ± 4.62f**	**6429.59 ± 5.34e**	**6413.78**	**-0.98**

*RC* Relative change, *Eta* Water consumptive use, *IWA* Irrigation water applied

It was observed that the amount IWA in the 2nd season was higher than that of the 1st one. The values ETa and IWA reached their peak under no priming (unsaline soil) and no priming (saline soil) treatments since they were 4875.35 and 6503.56 m^3^ ha^−1^, respectively, in the 2nd season (Table 5). The lowest values of ETa and IWA were attained under CaCl_2_ + SNP and SNP + CaCl_2_ treatments since they were 4776.76 and 6397.96 m^3^ ha^−1^, respectively, in the 1st season. The seeds priming agents could be arranged descendingly, following their effect on the Eta, in the following order: control > no priming > hydro > CaCl_2_ > SNP > CaCl_2_ + SNP > SNP + CaCl_2_, while IWA in the following order: no priming > hydro > control > CaCl_2_ > SNP > CaCl_2_ + SNP > SNP + CaCl_2_ (for both seasons).

### Crop water productivity (CWP) and irrigation water productivity (IWP)

CWP and IWP were affected by salinity and wheat grain priming in the winter season of 2019/20 and 2020/21, as presented in Table 6. The CWP and IWP were significantly increased due to the grain priming. The highest values obtained of CWP and IWP were 1.64 and 1.23 kg m^−3^, respectively, and were recorded at SNP + CaCl_2_ in the 2nd season. On the other hands, the lowest values of CWP and IWP were found to be 0.70 and 0.52 kg m^−3^, respectively, and recorded under no priming (saline soil) treatment in the 2nd growing season. The data from both seasons, on the basis of average, showed that CWP values were 1.51, 0.72, 0.96, 1.48, 1.51, 1.57 and 1.62 kg m^−3^, while IWP values were 1.14,0.54, 0.72, 1.10, 1.12, 1.17 and 1.21kg m^−3^ at control, No, hydro and CaCl_2_, SNP, CaCl_2_ + SNP and SNP + CaCl_2_, respectively. It was noticed that the amount of CWP and IWP was higher in the 2nd season than that of the 1st one in all treatments except the no priming in saline soil treatment (Table 6). The seeds priming could be arranged descendingly on the basis of their effect on the CWP and IWP in the following order: SNP + CaCl_2_ > CaCl_2_ + SNP > control > SNP > CaCl_2_ > hydro > no priming in saline soil for both seasons.

**Table 6:** Effect of Salinity and priming on wheat crop water productivity and irrigation water productivity

priming	CWP (kgm^−3^)		mean	RC	IWP (kgm^−3^)		mean	RC
	**2019/20**	**2020/21**			**2019/20**	**2020/21**		
**No priming (unsaline soil)**	**1.49 ± 0.21b**	**1.54 ± 0.17b**	**1.51**	**0.00**	**1.12 ± 0.16b**	**1.15 ± 0.19b**	**1.14**	**0.00**
**No priming (saline soil)**	**0.74 ± 0.07e**	**0.70 ± 0.13d**	**0.72**	**-56.93**	**0.56 ± 0.14d**	**0.52 ± 0.13d**	**0.54**	**-59.39**
**Hydro** **(H**_**2**_**O)**	**0.91 ± 0.22d**	**1.01 ± 0.21c**	**0.96**	**-2.02**	**0.68 ± 0.21c**	**0.75 ± 0.10c**	**0.72**	**-3.49**
**CaCl**_**2**_	**1.45 ± 0.04c**	**1.51 ± 0.03b**	**1.48**	**-2.02**	**1.08 ± 0.09b**	**1.13 ± 0.08b**	**1.10**	**-3.49**
**SNP**	**1.50 ± 0.06b**	**1.51 ± 0.04b**	**1.51**	**-0.21**	**1.12 ± 0.10b**	**1.12 ± 0.11b**	**1.12**	**-1.54**
**CaCl2 + SNP**	**1.52 ± 0.08b**	**1.62 ± 0.07a**	**1.57**	**3.83**	**1.14 ± 0.16ab**	**1.20 ± 0.22a**	**1.17**	**2.54**
**SNP + CaCl2**	**1.59 ± 0.11a**	**1.64 ± 0.09a**	**1.62**	**6.57**	**1.18 ± 0.10a**	**1.23 ± 0.10a**	**1.21**	**5.62**

*RC* Relative change, *CWP* Crop water productivity, *IWP* Irrigation water productivity

### Wheat traits and its yield

Wheat traits and their yield as affected by salinity and seeds priming in the winter season of 2019/20 and 2020/21 are presented in Tables (7&8).

#### Plant height

The plant height was significantly increased due to the seed priming. The highest value of plant height (108 cm) was recorded with SNP in the 2nd season. The lowest value of plant height (91.33 cm) was attained with no priming in saline soil in the 1st season. The data from both seasons, on the basis of average, Plant height recorded values were 103.88, 93.83, 97.83, 106.72, 107.98, 106.17 and107.33 cm at control, no priming in saline soil, hydro and CaCl_2_, SNP, CaCl_2_ + SNP and SNP + CaCl_2_, respectively. The data showed that the plant height was greater in the 2nd growing season than the first one in all treatments (Table 7). The seeds priming treatments, regarding their effect on the plant height, could be arranged descendingly in the following order: SNP > SNP + CaCl_2_ > CaCl_2_ > CaCl_2_ + SNP > control > hydro > no priming in saline soil, for both seasons.

**Table 7:** Effect of Salinity and seeds priming on wheat plant height and seed index

priming	plant height (cm)		mean	RC	seed index (g)		mean	RC
	**2019/20**	**2020/21**			**2019/20**	**2020/21**		
**No priming (unsaline soil)**	**103.34 ± 4.35c**	**104.43 ± 3.37c**	**103.88**	**0.00**	**41.1 ± 0.36e**	**41.59 ± 0.38e**	**41.36**	**0.00**
**No priming (saline soil)**	**91.33 ± 2.50e**	**96.33 ± 0.83e**	**93.83**	**-6.18**	**40.27 ± 0.53f**	**40.67 ± 0.68f**	**40.47**	**1.33**
**Hydro** **(H**_**2**_**O)**	**97.33 ± 3.50d**	**98.33 ± 3.88d**	**97.83**	**2.66**	**41.53 ± 0.66e**	**42.30 ± 0.34d**	**41.92**	**6.21**
**CaCl**_**2**_	**105.78 ± 0.92b**	**107.67 ± 0.14a**	**106.72**	**2.66**	**45.00 ± 0.62d**	**43.20 ± 0.49c**	**44.10**	**6.21**
**SNP**	**107.95 ± 0.97a**	**108.00 ± 0.54a**	**107.98**	**3.79**	**46.50 ± 0.35b**	**44.37 ± 0.11b**	**45.43**	**8.97**
**CaCl2 + SNP**	**105.67 ± 0.55b**	**106.67 ± 0.41b**	**106.17**	**2.16**	**45.67 ± 0.83c**	**44.63 ± 0.90b**	**45.15**	**8.39**
**SNP + CaCl2**	**107.00 ± 0.83a**	**107.67 ± 0.97a**	**107.33**	**3.22**	**48.33 ± 0.55a**	**46.80 ± 0.63a**	**47.57**	**13.05**

*RC* Relative change

##### Seed index

The seed index was significantly increased due to the seeds priming. The highest value of seed index (48.33 g) was recorded with SNP + CaCl_2_ in the 1st season. The lowest value of seed index (40.27g) was found with no priming in saline soil in the 1st season. On the basis of average from the two growing seasons, the obtained values of seed index were 41.36,40.47, 41.92, 44.10, 45.43, 45.15 and47.57g at control, no priming, hydro and CaCl_2_, SNP, CaCl_2_ + SNP and SNP + CaCl_2_, respectively. The seeds priming treatments, regarding their effect on the seed index, could be arranged descendingly in the following order: SNP + CaCl_2_ > SNP > CaCl_2_ + SNP > CaCl_2_ > hydro > control > no priming in saline soil, for both seasons.

##### Wheat grain yield

The grain yield was significantly increased due to the seeds priming. The highest value of grain yield (7.87 Mg ha^−1^) was recorded with SNP + CaCl_2_ in the 2nd season. The lowest value of grain yield (3.40Mg ha^−1^) was observed with no priming in the 1st season. On the basis of average from both seasons, the obtained values of grain yield were 7.38,3.50, 4.64, 7.12, 7.23, 7.51 and7.75 Mg ha^−1^ at control, no priming, hydro and CaCl_2_, SNP, CaCl_2_ + SNP and SNP + CaCl_2_, respectively. It was noticed in all treatments that the grain yield in the 2nd season was higher than the first one, as represented in Table 8. The seeds priming treatments from both seasons,could be arranged descendingly regarding their effect on the grain yield according to the following order: SNP + CaCl_2_ > CaCl_2_ + SNP > control > SNP > CaCl_2_ > hydro > no priming in saline soil.

##### Wheat straw yield

The straw yield was significantly increased due to the seeds priming. The highest value of straw yield (10.74Mg ha^−1^) was recorded with SNP + CaCl_2_ in the 1st season. The lowest value of straw yield (6.62Mg ha^−1^) was found with no priming in the 2nd season. On the basis of average from both growing seasons, the obtained values of straw yield were10.64, 6.96, 8.91, 9.84, 9.85, 10.48 and10.65 Mg ha^−1^ at control, no priming, hydro and CaCl_2_, SNP, CaCl_2_ + SNP and SNP + CaCl_2_, respectively (Table 8). The seeds priming could be arranged descendingly according to their effect on the straw yield in the following order: SNP + CaCl_2_ > control > CaCl_2_ + SNP > SNP > CaCl_2_ > hydro > no priming in saline soil, for both seasons.

**Table 8:** Effect of Salinity and seeds priming on wheat grain and straw yield

priming	Grain yield (Mg ha^−1^)		mean	RC	Straw yield (Mg ha^−1^)		mean	RC
	**2019/20**	**2020/21**			**2019/20**	**2020/21**		
**No priming (unsaline soil)**	**7.25 ± 0.83b**	**7.51 ± 0.74b**	**7.38**	**0.00**	**10.67 ± 0.71a**	**10.61 ± 0.62a**	**10.64**	**0.00**
**No priming (saline soil)**	**3.40 ± 0.33e**	**3.60 ± 0.62f**	**3.50**	**-59.05**	**7.29 ± 0.50f**	**6.62 ± 0.24e**	**6.96**	**-19.48**
**Hydro** **(H**_**2**_**O)**	**4.40 ± 0.32d**	**4.88 ± 0.44e**	**4.64**	**-3.63**	**8.68 ± 0.33e**	**9.13 ± 0.43d**	**8.91**	**-8.09**
**CaCl**_**2**_	**6.94 ± 0.12c**	**7.30 ± 0.14d**	**7.12**	**-3.63**	**9.72 ± 0.16c**	**9.97 ± 0.15c**	**9.84**	**-8.09**
**SNP**	**7.20 ± 0.15b**	**7.25 ± 0.21d**	**7.23**	**-2.12**	**9.34 ± 0. 4d**	**10.35 ± 0.12b**	**9.85**	**-8.17**
**CaCl2 + SNP**	**7.28 ± 0.14b**	**7.75 ± 0.07c**	**7.51**	**1.78**	**10.34 ± 0.12b**	**10.61 ± 0.17a**	**10.48**	**-1.57**
**SNP + CaCl2**	**7.62 ± 0.16a**	**7.87 ± 0.08a**	**7.75**	**4.73**	**10.74 ± 0.11a**	**10.55 ± 0.19a**	**10.65**	**0.05**

*RC* Relative change

##### Grain nitrogen content

Grain nitrogen content as affected by salinity and seeds priming in the first season of 2019/20 and the second season 2020/21 is represented in Table 9. The grain nitrogen content was significantly increased due to the seeds priming. The value of nitrogen content in grain reached its peak (2.90%) with SNP + CaCl_2_ during 2nd season. The lowest value of grain nitrogen content (2.23%) was observed with no priming in the 2nd season. On the basis of average from both growing seasons, values of nitrogen content in grain were 2.39, 2.29, 2.49, 2.56, 2.61, 2.69 and2.88% at control, no priming, hydro and CaCl_2_, SNP, CaCl_2_ + SNP and SNP + CaCl_2_, respectively. The seeds priming, regarding their effect on grain nitrogen content, could be arranged descendingly in the following order: SNP + CaCl_2_ > CaCl_2_ + SNP > SNP > CaCl_2_ > hydro > control > no priming, for both seasons.

**Table 9:** Effect of Salinity and seeds priming on wheat grain nitrogen content

priming	Nitrogen of grain (%)		mean	RC
	**2019/20**	**2020/21**		
**No priming (unsaline soil)**	**2.42 ± 0.03d**	**2.3 ± 0.06d**	**2.39**	**0.00**
**No priming (saline soil)**	**2.35 ± 0.07d**	**2.23 ± 0.09e**	**2.29**	**3.82**
**Hydro** **(H**_**2**_**O)**	**2.52 ± 0.04c**	**2.45 ± 0.03c**	**2.49**	**6.76**
**CaCl**_**2**_	**2.63 ± 0.02b**	**2.50 ± 0.06c**	**2.56**	**6.76**
**SNP**	**2.57 ± 0.04c**	**2.64 ± 0.03b**	**2.61**	**8.25**
**CaCl2 + SNP**	**2.67 ± 0.08b**	**2.71 ± 0.08b**	**2.69**	**11.15**
**SNP + CaCl2**	**2.86 ± 0.06a**	**2.90 ± 0.04a**	**2.88**	**17.01**

*RC* Relative change

### Shoot and root growth

**Fig. 1:**
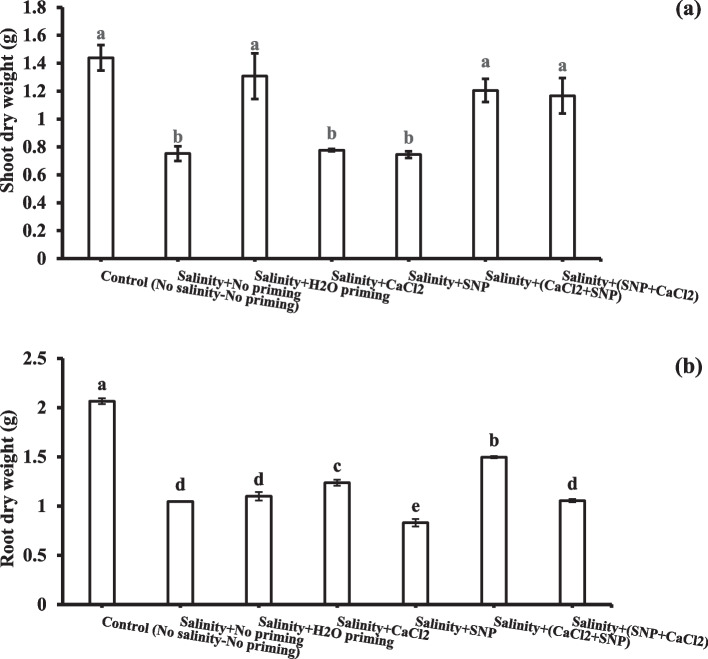
Dry weight of shoot (**a**) and root (**b**) as affected by salinity stress and seeds priming application (calcium chloride CaCl_2_, sodium nitroprusside SNP, and hydropriming H_2_O). Litters on bars indicate significance level of difference according to Duncan's test at *p* < 0.05

The data represented in Fig. 1 a and b of shoot and root dry weight showed the significant inhibitory effect of salinity on wheat plant growth and dry matter gain. Shoot dry matter of wheat plants treated with hydro-priming or successive chemical priming agents (CaCl_2_ + SNP & SNP + CaCl_2_) counteracted salinity stress effect significantly and preserved growth rates up to control. On the other hand, exogenous application of osmo-priming (calcium chloride) individually or successively with sodium nitroprusside as redox priming (CaCl_2_ + SNP) resulted in a significant increase in root dry weight compared to corresponding salinity stressed plants without priming treatments.

### Photosynthetic pigments

**Fig. 2:**
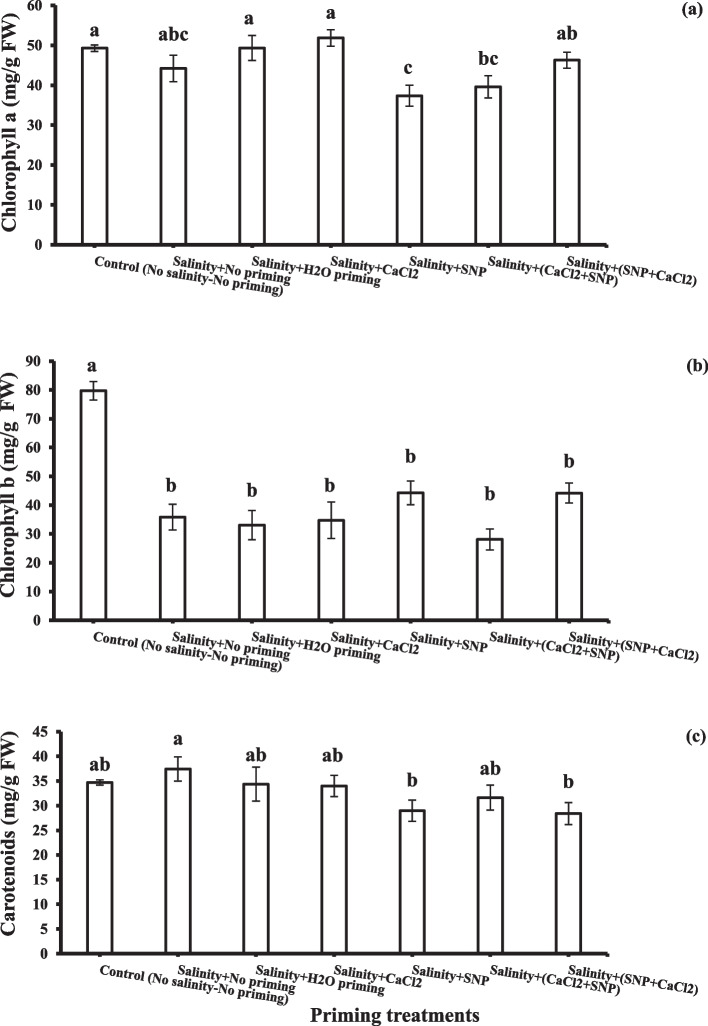
photosynthetic pigments Chl.a, (**a**) Chl.b (**b**) and carotenoids (**c**) as affected by salinity stress and seeds priming application (calcium chloride CaCl_2_, sodium nitroprusside SNP, and hydropriming H_2_O). Litters on bars indicate significance level of difference according to Duncan's test at *p* < 0.05

The biosynthesis of photosynthetic pigments (Chl. a, Chl. b and carot.) in the leaves of wheat plants that were grown after seed priming under salinity stress was analyzed and represented in Fig. 2a, b and c. Generally, Salinity did not significantly affect chlorophyll a contents and carotenoids, while chlorophyll b reduced significantly under salinity stress. Also, it was observed that chlorophyll a and carotenoids contents of wheat plants that were treated with hydro-priming or individual osmo-primin (CaCl_2_) were improved equal to or higher than those of the control plants. Slight induction in chl.a content was observed due to successive SNP + CaCl_2_ application.

### Sodium and potassium

**Fig. 3:**
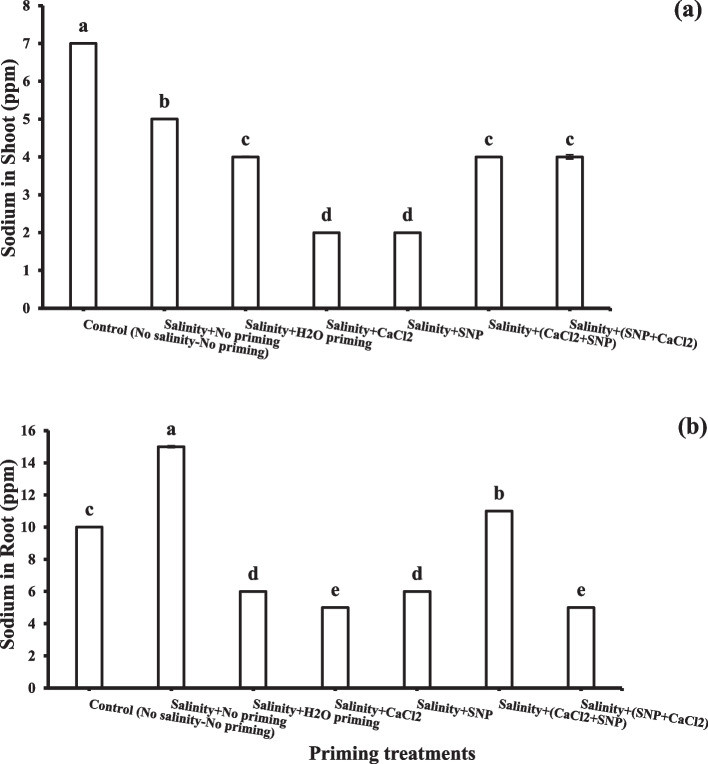
Sodium (Na.^+^) concentration in shoots (a) and roots (b) as affected by salinity stress and seeds priming application (calcium chloride CaCl_2_, sodium nitroprusside SNP, and hydropriming H_2_O). Litters on bars indicate significance level of difference according to Duncan's test at *p* < 0.05

It was observed that all priming treatments diminished shoot sodium contents under soil salinity stress (Fig. 3a). The lowest value (2 ppm) of sodium content in the shoot was recorded with individual osmo-priming (CaCl_2_) and SNP. A similar trend was observed in roots under salinity stress (Fig. 3b), where all the priming treatments decreased the sodium content in the roots except for successive priming with CaCl_2_ + SNP, which showed the highest root sodium content (11 ppm).

**Fig. 4:**
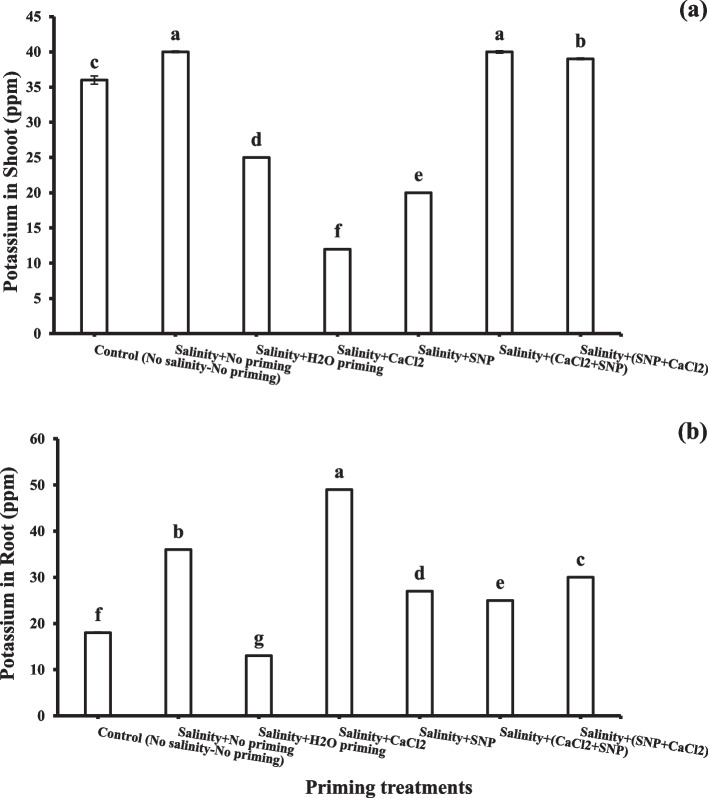
Potassium (K +) concentration in shoots (**a**) and roots (**b**) as affected by salinity stress and seeds priming application (calcium chloride CaCl_2_, sodium nitroprusside SNP, and hydropriming H_2_O). Litters on bars indicate significance level of difference according to Duncan's test at *p* < 0.05

Potassium content in shoots decreased under salinity stress (Fig. 4a). It was observed that successive priming with CaCl_2_ + SNP and SNP + CaCl_2_ enhanced potassium contents in shoot (40 ppm and 39 ppm, respectively). On the other hand, all priming treatments enhanced potassium contents in roots under salinity stress (Fig. 4b). The highest potassium content in the root was recorded with individual osmo-priming CaCl_2_ (49 ppm).

### Malondialdehyde MDA

**Fig. 5:**
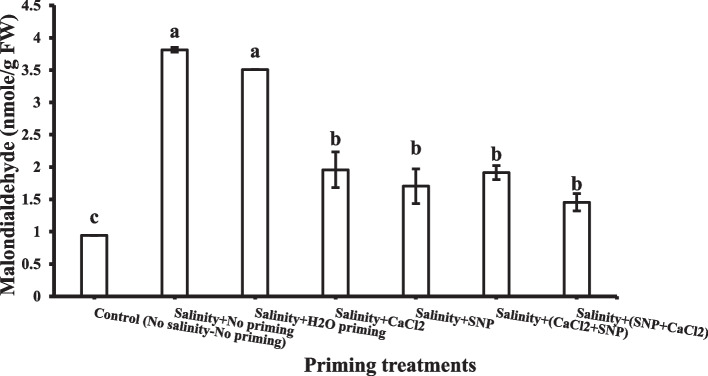
Shoot Malondialdehyde (MDA) as affected by salinity stress and seeds priming application (calcium chloride CaCl_2_, sodium nitroprusside SNP, and hydropriming H_2_O). Litters on bars indicate significance level of difference according to Duncan's test at *p* < 0.05

Malondialdehyde (MDA) contents in shoots were increased under salinity stress. All priming treatments significantly reduced MDA content in shoot except for hydro-priming, which showed a high MDA content in shoot (Fig. 5).

Exogenous SNP and CaCl_2_ application as priming agents (individually or successively) reduced the concentrations of MDA within the wheat shoot system exposed to salinity.

### Proline

**Fig. 6:**
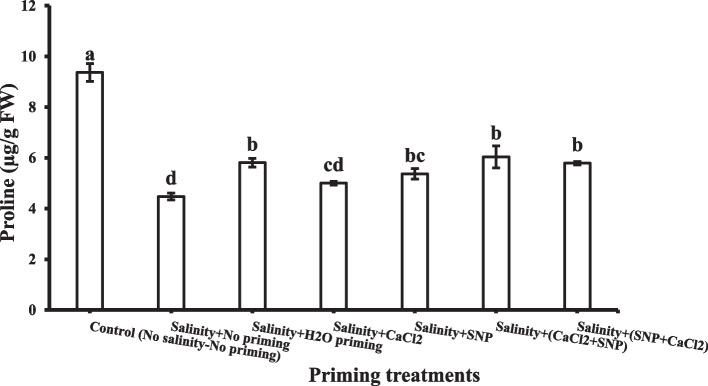
Shoot proline as affected by salinity stress and seeds priming application (calcium chloride CaCl_2_, sodium nitroprusside SNP, and hydropriming H_2_O). Litters on bars indicate significance level of difference according to Duncan's test at *p* < 0.05

Proline contents in shoots decreased under salinity stress (Fig. 6). All priming treatments enhanced proline contents in the shoot; their effect on proline in the shoot could be arranged in ascending order as following: CaCl_2_ < SNP < SNP + CaCl_2_ < H_2_O < CaCl_2_ + SNP. Successive priming with CaCl_2_ + SNP induced the highest proline contents in shoot (6 µg/ g FW).

#### Phenolics

**Fig. 7:**
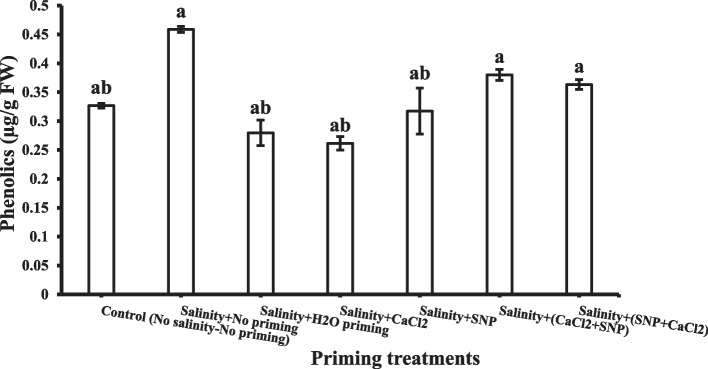
Shoot phenolics contents as affected by salinity stress and seeds priming application (calcium chloride CaCl_2_, sodium nitroprusside SNP, and hydropriming H_2_O). Litters on bars indicate significance level of difference according to Duncan's test at *p* < 0.05

In general, all priming treatments could not change the contents of phenolics in shoot significantly under salinity stress (Fig. 7). Among priming treatments, successive priming with CaCl_2_ + SNP and SNP + CaCl_2_ showed the highest value of phenolics contents in shoot (0.38 and 0.36 µg/ g FW, respectively).

#### Total antioxidants (DPPH)

**Fig. 8:**
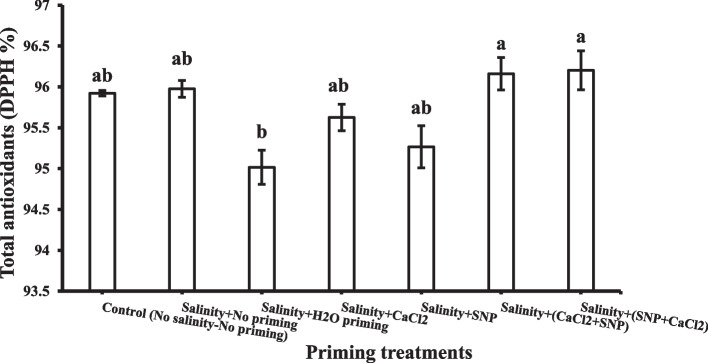
Shoot total antioxidants (DPPH) as affected by salinity stress and seeds priming application (calcium chloride CaCl_2_, sodium nitroprusside SNP, and hydropriming H_2_O). Litters on bars indicate significance level of difference according to Duncan's test at *p* < 0.05

Successive priming with SNP + CaCl_2_ and CaCl_2_ + SNP recorded the highest value of total antioxidants contents in shoot under salinity stress (Fig. 8).

#### Enzymatic antioxidants

**Fig. 9:**
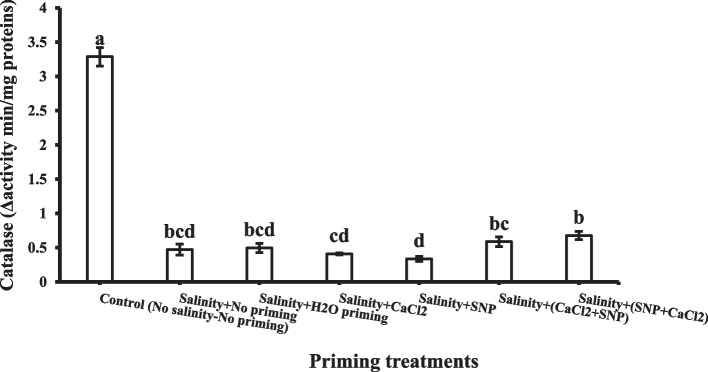
Shoot Catalase enzyme (CAT) as affected by salinity stress and seeds priming application (calcium chloride CaCl_2_, sodium nitroprusside SNP, and hydropriming H_2_O). Litters on bars indicate significance level of difference according to Duncan's test at *p* < 0.05

**Fig. 10:**
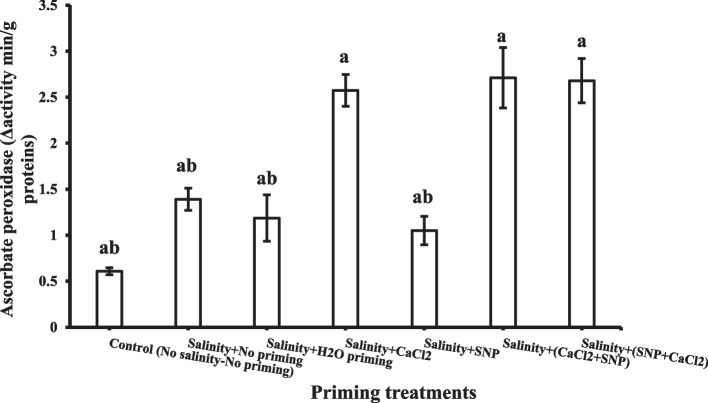
Shoot Ascorbate peroxidase enzyme (APX) as affected by salinity stress and seeds priming application (calcium chloride CaCl_2_, sodium nitroprusside SNP, and hydropriming H_2_O). Litters on bars indicate significance level of difference according to Duncan's test at *p* < 0.05

Catalase enzyme activities in wheat plant shoots decreased significantly under salinity stress. The priming treatments slightly improved catalase enzyme activities (Fig. 9). While ascorbate peroxidase activities in shoots were increased under most priming treatments under salinity stress (Fig. 10), their effect upon ascorbate peroxidase activities in the shoot could be arranged in ascending order as following: SNP < H_2_O < CaCl_2_ < SNP + CaCl_2_ < CaCl_2_ + SNP. It was detected that successive priming with CaCl_2_ + SNP, SNP + CaCl_2_ and individual CaCl_2_ showed the highest value of ascorbate peroxidase activity in the shoot.

#### Soluble proteins

**Fig. 11:**
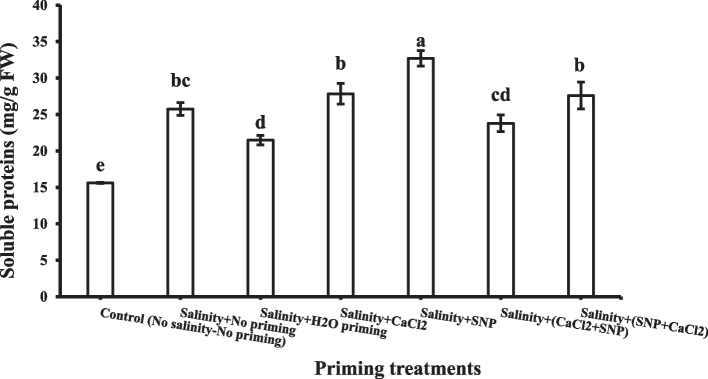
Shoot soluble proteins as affected by salinity stress and seeds priming application (calcium chloride CaCl_2_, sodium nitroprusside SNP, and hydropriming H_2_O). Litters on bars indicate significance level of difference according to Duncan's test at *p* < 0.0

All priming treatments under salinity stress increased the soluble proteins contents in shoot significantly (Fig. 11). Their effect on soluble proteins contents in the shoot could be arranged in ascending order as following: H_2_O < CaCl_2_ + SNP < SNP + CaCl_2_ < CaCl_2_ < SNP. Individual priming with SNP showed the highest value of soluble proteins contents in the shoot (32.7 mg/ g FW).

#### Principle Component analysis (PCA)

**Fig. 12:**
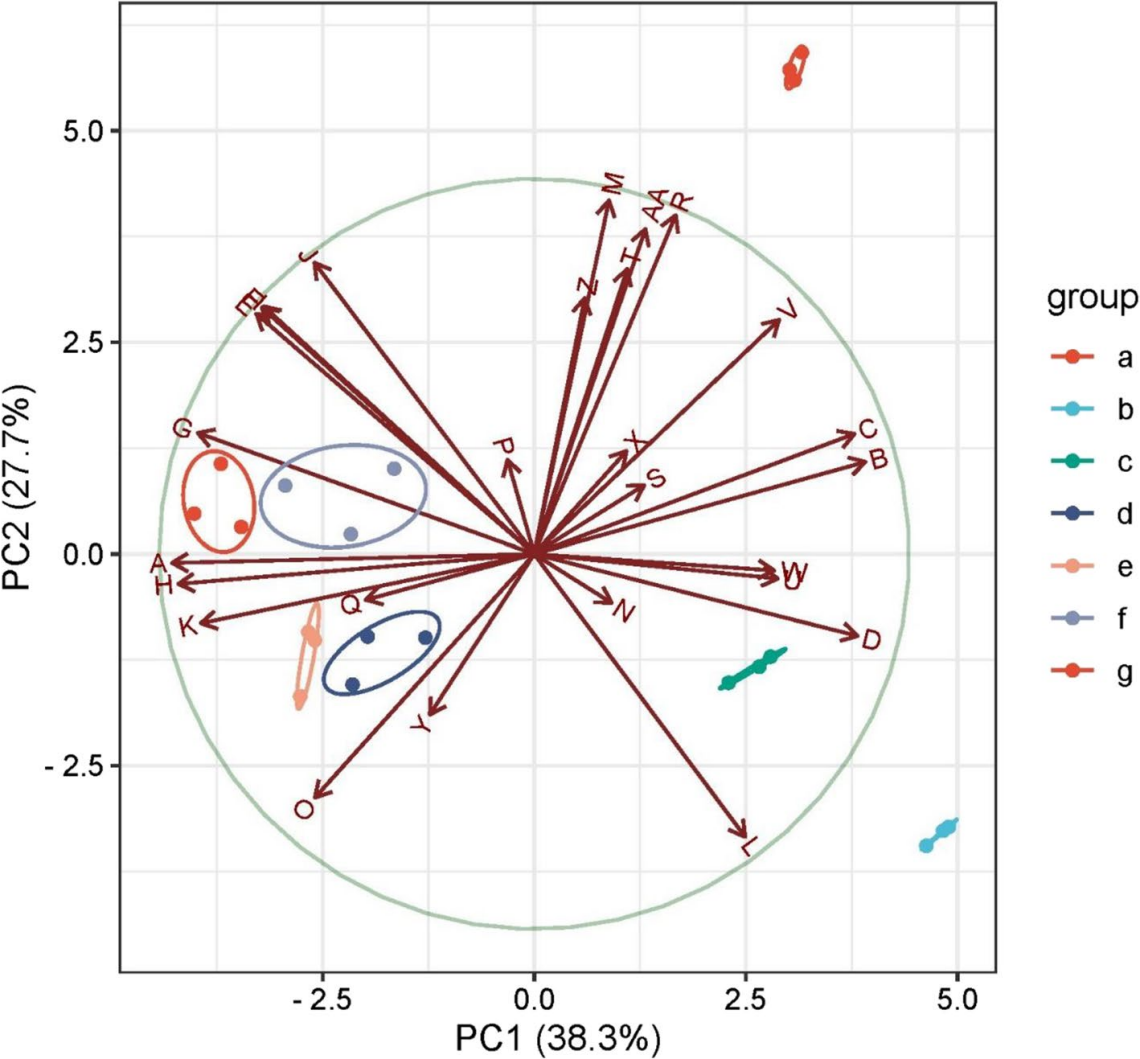
Loading plot of different studied attributes under salinity stress correlations to the first two Principal Component analysis (PCA) axes, Horizontal and vertical arrows indicate the rise-direction of salinity and priming treatments. a = control, b = salinity without priming, c = salinity + hydro priming, d = salinity + CaCl_2_ priming, e = salinity + SNP priming, f = salinity + (CaCl_2_ + SNP) priming, g = salinity + (SNP + CaCl_2_) priming. parameters: A = hydraulic conductivity (HC), B = Bulk desity (Bd), C = water consumptive use (Eta), D = irrigation water applied (IWA), E = Crop water productivity (CWP), F = irrigation water productivity (IWP), G = Plant height, H = Seed index, I = Grain yield, J = Straw yield, K = Nitrogen content, L = MDA, M = Proline, N = Phenolics, O = protein, *P* = total antioxidants DPPH, Q = ascorpate peroxidase APX, R = catalase CAT, S = chlorophyll a, T = chlorophyll b, U = carotenoids, V = Na^+^shoot, W = Na^+^root, X = K^+^shoot, Y = K^+^root, Z = Shoot D.Wt., AA = Root D.Wt

Subjecting the original data of all assessed traits to the analysis of the principle component (PCA, Fig. 12) gives clear details for all possible negative and positive correlations among all measured traits. Thus, the PCA biplot indicated great contrariness between Eta, IWA and Bd (the right-hand half of Fig. 12) and the growth in addition to yield indicators (the left-hand half). PCA axis 1 captures about 38.3% of the cumulative percentage, followed by the second axis (27.7%). The right-hand half of Fig. 12 was greatly affected by the following treatments: control (No salinity-No priming), no priming in saline soil and salinity + hydro-priming. Meanwhile, the left-hand half was greatly affected with salinity + CaCl_2_ priming, salinity + SNP priming, salinity + (CaCl_2_ + SNP) priming, and salinity + (SNP + CaCl_2_) priming treatments. On the first PCA axis, strong positive correlations were found among CWP & IWP and APX, proteins and ions (K^+^ root) as well as growth parameters (plant height). Those are also positively correlated with different productivity attributes such as seed index, straw yield. All of these traits were arranged on the left-hand side half of PCA correlation biplot. Meanwhile, they were negatively correlated with IWA, Bd, Eta, antioxidants (CAT, proline, & free phenolics), stress marker (MDA) and ions (Na^+^root & shoot, K^+^ shoot) and growth parameters such as dry weight of shoots & roots and photosynthetic pigments (ch.a, ch.b &carot). Second PCA axis showed another direction of trait correlation, i.e. some of the assessed growth and productivity parameters (root and shoot dry weight, plant height and straw yield) were arranged in the upper side half of the PCA correlation biplot and positively correlated with CWP, IWP, proline, CAT, total antioxidants, and K^+^ shoot. Meanwhile, they were negatively correlated with soluble proteins, K^+^ root, nitrogen content, MDA, and enzymatic and non-enzymatic antioxidants such as APX and phenolics. The quasi-trend of the assessed growth parameters showed clear negative correlations with MDA and Na^+^ in the root. Most of the determined growth parameters (root and shoot dry weight, Ch.a, Ch.b) were positively correlated with K^+^ and Na^+^ in shoot, proline, CAT, Bd and Et;, all of them occupied the upper left-hand quadrate of the PCA biplot..

#### Correlation analysis

**Fig. 13:**
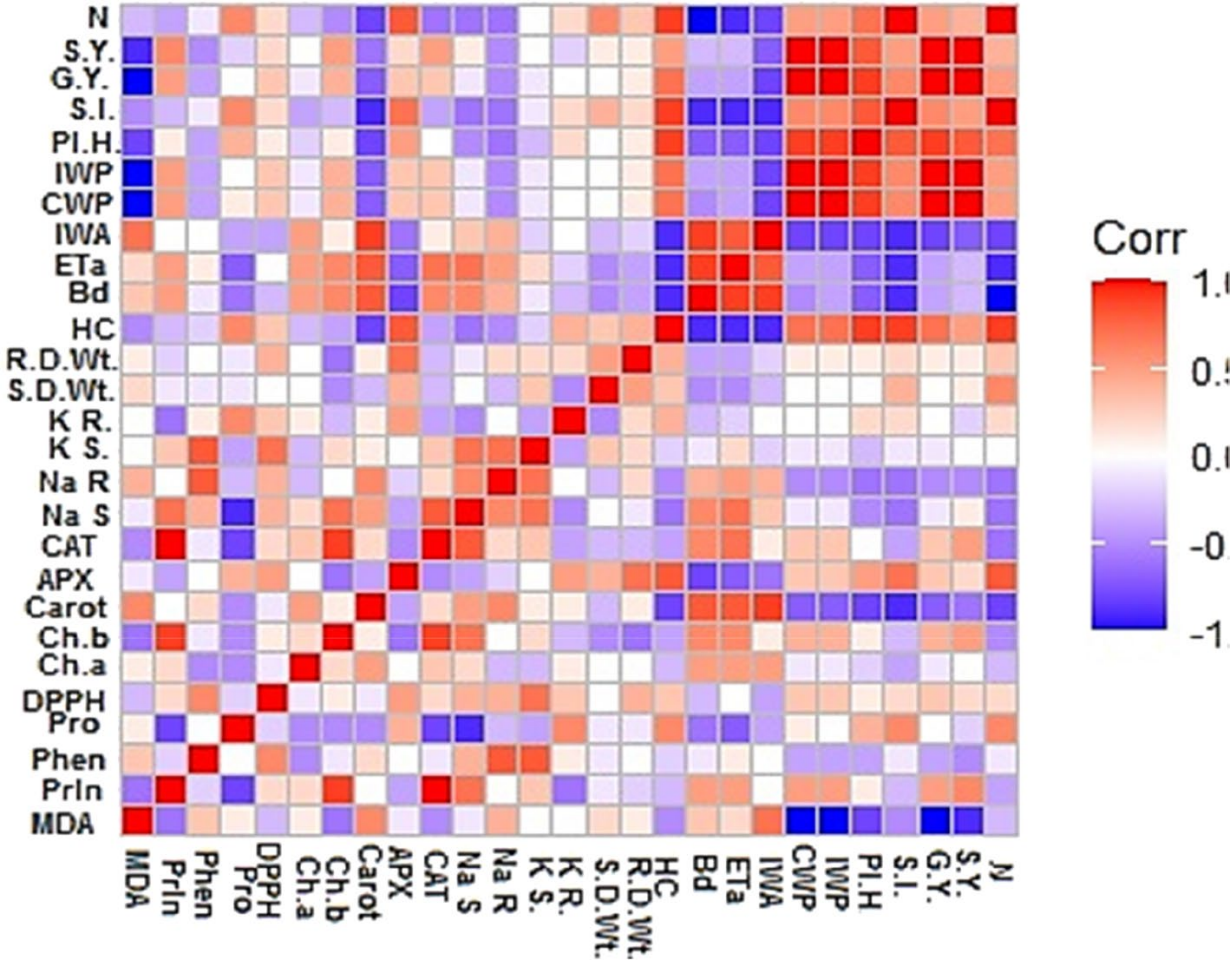
Correlation matrix of the 27 measured traits of the studied parameters in shoot and root of wheat plants with priming agents (calcium chloride CaCl_2_, sodium nitroprusside SNP, and hydropriming H_2_O) under salinity stress. The increasing color intensities illustrate a higher correlation coefficient. parameters: N=nitrogen content, S.Y.= Straw yield, G.Y.=grain yield, S.I.=Seed index, Pl.H.=plant height, IWP=irrigation water productivity, CWP=crop water productivity, IWA=irrigation water applied, Eta=water consumptive use, Bd=bulk density, HC=hydraulic conductivity, R.D.Wt=root dry weight, S.D.Wt=shoot dry weight, KR=K^+^root, KS=K^+^shoot, Na R=Na^+^ Root, Na S=Na^+^ shoot, CAT= catalase, APX=ascorbate peroxidase, Carot.=carotenoids, Ch.a=chlorophyll a, Ch.b= chlorophyll b, DPPH= total antioxidants, Pro= proteins, Phen=phenolics, Prln=proline

A visual plot of correlation analysis is used to find positive and negative correlations among multiple parameters under different treatments (Fig. 13). Strong negative correlations were observed between Bd, Eta, IWA from one side and SY, GY, CWP, IWP, SI, plant height and nitrogen content in grains from the other side. Another negative correlation can be observed between MDA from one side and CWP, IWP, SI and GY from the other side. A strong positive correlation could be noticed among all these parameters (SY, GY, CWP, IWP, SI, plant height and nitrogen content in grains). Another positive correlation was seen among IWA, Eta, and Bd.

#### Heat map analysis

**Fig. 14:**
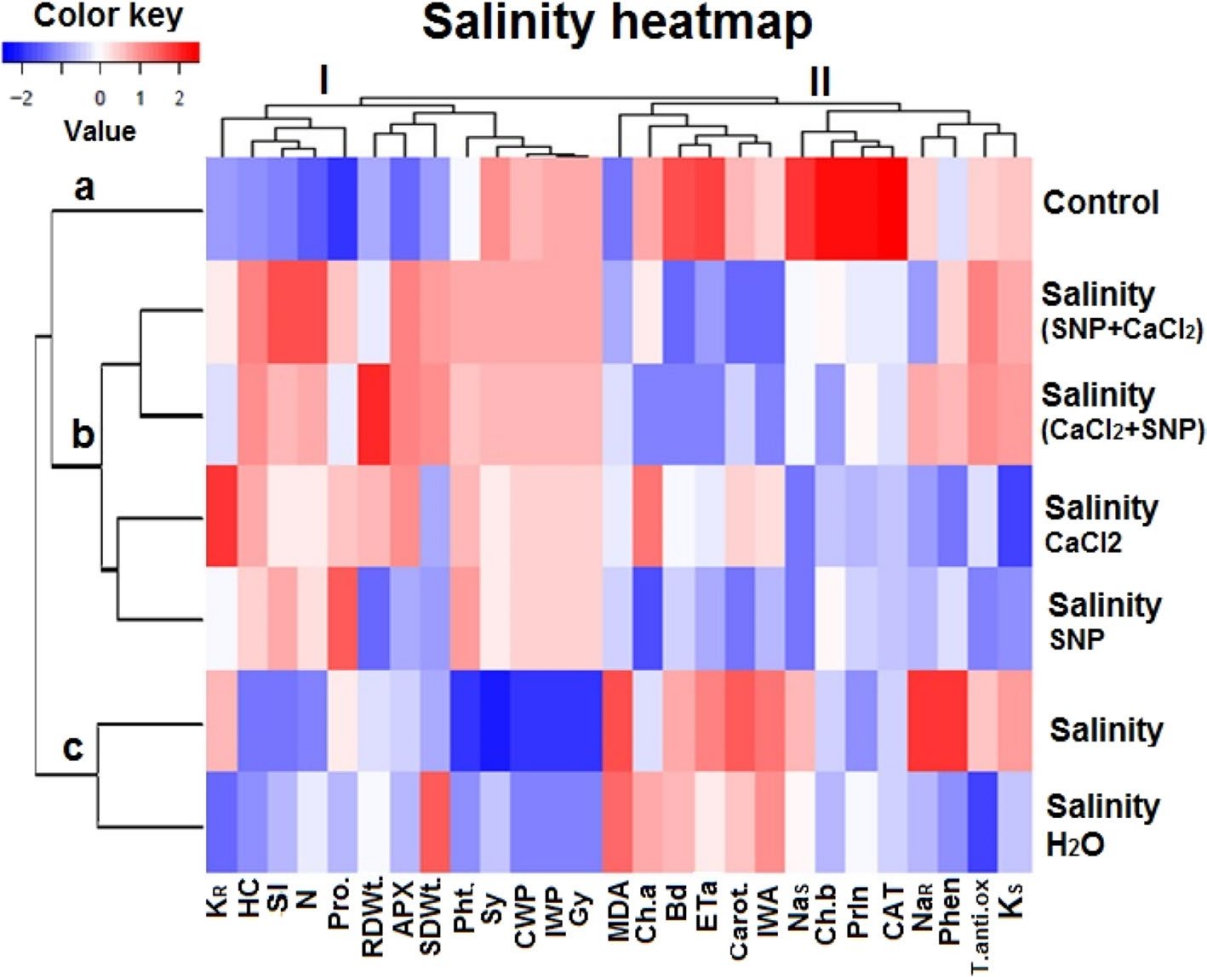
Loading plot of different studied attributes under salinity stress heatmap

As observed in Fig. 14, hierarchical clustering analysis and a heat map clearly identified the significant differences between treatments on the left side and parameters on top. Priming agents CaCl_2_ and SNP application individually or successively changed the response of all studied growth, physiological and yield attributes under salinity stress compared to salinity treatment without priming (Fig. 14). It was observed that growth and yield attributes clustered with antioxidant enzyme APX and proline, as observed in the heatmap and hierarchical cluster analysis (HCA) in Fig. 14.

## Discussions

Through the higher plant life cycle, seed germination is considered the most decisive phase. A plethora of biochemical and physiological processes are activated inside seeds after rehydration, and water becomes sufficiently favorable for different metabolic activities, including respiration and protein synthesis [60]. Nevertheless, under salinity stress, germination performance is hindered by toxicity of Na^+^ and Cl^−^, resulting in osmotic potential and ROS production [61]. The role of nitric oxide and calcium application in physiological processes is intensively reviewed in the literature [26, 32]. However, little data is available on the effect of nitric oxide priming individually or in combination with calcium under field conditions.

The data revealed a reduction in the amount of water consumptive use (ETa) and irrigation water applied (IWA) with seeds priming. The lowest values of ETa and IWA were attained under CaCl_2_ + SNP and SNP + CaCl_2_ treatments. Semize tal. [62] reported that the soil salinity affects ETa due to the ion-specific toxicity and the decrease in both available water and photosynthetic activity. A similar trend was reported by Zhang et al. [63] who found that salinity treatments reduced ETa values in comparison with treatment without salinity application. Also, the reduction ratio of the yields was less than that of ETa.

In the present study seeds priming increased the CWP and IWP, with the highest values obtained at successive SNP + CaCl_2_ in the 2nd season. Improving CWP can be achieved by increasing the production per unit of water consumed, or reducing the amount of water consumed per unit yield of production [64]. Increasing levels of subsoil NaCl salinity significantly depressed the water uptake with a depressing effect on water use efficiency. Also, there was a 21% decline in the water use efficiency of wheat when subsoil NaCl salinity was increased from S1 to S5 [65]. Conditions inducing stomatal closure, such as water stress and salinity, restrict the CO_2_ supply to carboxylation sites that increases the intrinsic water use efficiency of the plant [66]. At Luancheng station, and based on field experiments during the period from 1987 to 2015, it was recorded that the average of CWP in winter wheat ranged from 1.36 to 2.07 kg m^−3^ [63]. Also, soil salinity affects CWP due to ion-specific toxicity and decrease both available water and photosynthetic activity [62].

The data obtained in this study revealed the positive effect of CaCl_2_ and NO on alleviating salt stress on the water relations of wheat plants. During drought and salinity stress, water utilization is one of the most affected mechanisms of the plants [67]. Exogenous application of SNP improves water budgeting, leaf turgor and osmotic potentials of wheat plant under drought stress [68]. Relative water content, soluble sugar accumulation, and osmolyte were increased by CaCl_2_ treatment for wheat genotypes under stress [69].

The data obtained herein revealed that seed priming enhanced plant height, seed index, grain yield, and straw yield. Successive SNP + CaCl_2_ achieved the highest records. While Plant height, grain yield, and yield components were reduced significantly with the application of salinity. It has been previously documented that salinity stress causes plants to be exposed to three major challenges, including increasing osmotic pressure, misbalancing ion uptake, and oxidative stress [70]. Salinity stress induces the closure of stomata and a reduction in leaf expansion rate, which in turn restricts plant growth and yield production [71]. The increase in salt concentration in plant growth media severely reduces germination rate, seedling establishment, growth, development, and survival, which are critical parameters in determining plant productivity [72]. Under salinity stress, the wheat crop exhibits a slower growth rate, reduced tillering, and reduced grain yield [73]. In the winter wheat, Zhang et al. [63] detected that the yields average changed from 4160.7 to 7000.9 kg ha^−1^ during 1987–2015, based on field experiments at Luancheng station. Salinity stress reduced grain yields less than those without salinity. Elevated salt concentration in the growth medium imposes strong deleterious impacts on plant biomass [73]. Plant physiological functioning is negatively affected with soil salinity, which resulted in a major fraction of photosynthesis that divert or counter the negative effects of salinity instead of plant growth and development [74]. The exposure of seeds to salinity inhibits water imbibition, which in turn negatively affects the germination of seeds [75]. The improvement recorded in this study in plant height, seed index, the grain yield and straw yield as a result of seed priming treatments, particularly successive SNP + CaCl_2_, could be attributed to improved water relations and many biochemical alterations that consequently induced significant enhancement in wheat biomass and productivity under salt stress conditions. The enhancement of yield parameters indicates the activation of stress memory due to successive SNP + CaCl_2_ priming treatment, which provoked salt stress resistance until the maturity of the wheat plant. Many previous studies regarding seed priming with SNP and CaCl_2_ supported our finding under drought and salt stress [31, 37, 76].

This study showed that grain nitrogen content is negatively affected by salinity. Meanwhile, grain nitrogen content increased significantly due to seeds priming, particularly successive SNP + CaCl_2_ during the 2nd season. A previous study reported that salinity reduced the contents of grain fats, proteins, and fibers significantly. According to Ashraf and Harris [77], salt tolerant cultivars of rice, sunflower, barley, and finger millet showed a higher content of soluble proteins. Maqsood et al. [78] reported that salt stress caused a reduction in the accumulation of fiber and protein content in maize grain. Similar findings are given by [79, 80].

The data from this study showed the negative effect of salinity on wheat dry matter gain, while calcium chloride individually or successively CaCl_2_ + SNP, significantly increased root dry weight. Our results are in accordance with the postulated hypothesis that reporting salinity stress can negatively disrupt the performance and normal functioning of plants by hampering plant growth in addition to biochemical processes [81]. The negative effects of salinity stress arise from reducing water retention and cell turgidity, the closure of stomata, and ultimately hampering plant growth and yield [82]. Salinity stress imparted a significant effect on plant vegetative stage and reduced dry biomass. The aforementioned negative effects of salinity stress on plant growth parameters may be attributed to the excessive accumulation of Na and Cl ions around the root system and accordingly, the toxic effect in plant cells. This result is in harmony with those obtained by Abdel Latef and Chaoxing, [83] on pepper and Mostofa et al. [84] on rice. The exogenous application of successive priming treatment (CaCl_2_&SNP) might help plants cope with the negative effects of salinity stresses through revamping of biochemical processes. The addition of SNP remarkably amended plants shoot and root growth under salt stress.

Changes in photosynthetic pigments are important for determining the level of plant stress tolerance. Many researchers have shown that salt stress leads to the disturbance of ion homeostasis with the excessive accumulation of toxic ions, which causes a great deleterious effect on critical metabolic processes like water status, nutrient uptake, photosynthetic pigments and efficiency [85, 86]. Therefore, hydro-priming or osmo-priming seeds with CaCl_2_ can be an effective and environmentally friendly tool to enhance crop resistance. Moreover, seed priming with CaCl_2_, when compared to crop spray, can offer great economic advantages as it can easily be applied by growers and seed distributors. Calcium exogenous application can increase the concentration of Ca^2+^ in plants, particularly through resistance activation [30]. A slight induction in chl.a content was observed due to successive SNP + CaCl_2_ application, which could be attributed to the dual effect of Ca^2+^ and SNP. Increased photosynthetic pigments due to nitric oxide (SNP) had previously been reported in salt-stressed plants [14, 87].

All priming treatments was observed to reduce shoot sodium contents under soil salinity stress. Gupta et al. [87] reported that toxic accumulation of Na^+^ ions triggered by salinity leads to the generation of ROS, which may further disturb the homeostasis of cellular redox. Due to the higher accumulation of Na^+^ in shoots than in roots, leaves are more vulnerable to Na^+^ than roots [88]. Sodium (Na^+^) is transported up to shoots through the rapid movement of the xylem transpiration stream, but Na^+^ can return to roots only via the phloem. Regarding the high recirculation of Na^+^ from shoot to root, there is very limited evidence, suggesting the transport of Na^+^ is mostly unidirectional and results in high Na^+^ accumulation as leaves age. The Na^+^-specific effects are superimposed on the osmotic effects of NaCl and, importantly, show greater variation within species than the osmotic effect. Na^+^-specific damage is associated with the accumulation of Na^+^ in leaf tissues and results in the necrosis of older leaves. The disruption in protein synthesis appears to be an important cause of damage by Na^+^ [89]. Many previous studies reported that SNP, under salinity stress, stimulated the expression of the plasma membrane H^+^-ATPase, indicating a NO role in sustaining a higher K^+^/Na^+^ ratio [90]. According to Shaki et al. [91], redox priming with SA mitigated salinity adverse effect by enhancing the ability of cell membrane in root to hinder and reduce the entry of harmful ions, such as Cl‾ and Na^+^. Salinity negative effect was alleviated by SA application through up-regulation of Na^+^/H^+^ antiporters (NHX1and SOS1) along with ion homeostasis regulation. This shows a comprehensive role of redox priming in mitigating salinity stress which can be used as a successful model for salinity tolerant cultivation [92]. 

Maintaining the ratio of K^+^/Na^+^ in cellular compartments has been closely correlated with Salt tolerance, and correlated to low accumulation of Na^+^ [93]. Concordantly, NO treatment reduced the toxic content of Na^+^ ions in wheat plants under salinity stress. In addition, exogenous application of NO has been reported to induce the expression of H^+^-PPase and H^+^-ATPase. As a secondary messenger, NO treatment can activate secondary transporters to generate a very powerful electrochemical potential gradient and increase the exchange activity of Na^+^/H^+^ [94].

Salinity stress negatively affected potassium content in wheat plants, while successive priming with CaCl_2_ + SNP and SNP + CaCl_2_ enhanced shoot potassium contents. Also, all priming treatments under salinity stress enhanced potassium contents in root. Potassium (K^+^) has been reported to play a role in salinity stress remediation and osmotic stress, and many previous studies reported the restriction of K^+^ influx by sodium chloride NaCl [95]. Tester and Davenport, [89] reported that high Na^+^ concentration hampers the uptake of other nutrients by (1) interfering with transporters in the root plasma membrane, such as K^+^-selective ion channels, and (2) reducing root growth by high Na^+^ concentration.

Sodium Na^+^ can compete directly for K^+^- binding sites on enzymes, suggesting that the cytosolic K^+^ to Na^+^ ratio, rather than the absolute Na^+^ concentration, is critical for tolerance [96]. Calcium (Ca^+2^) protective effect in salt-affected plants could be connected to its role in membrane integrity maintenance, because one of the salinity negative effects is membrane integrity disruption caused by displacing Ca^+2^ions from the cell surface by Na^+^ ions [97]. The results showed that CaCl_2_ priming could sustain K^+^ intake under salinity stress. A external priming agent (CaCl_2_) could enhance Ca^+2^ content, thus increasing K^+^ influx.

Many previous studies reported the positive effects of exogenous NO. The role of NO has been attributed to ionic homeostasis regulation (particularly K^+^/Na^+^), activating antioxidant systems and restricting oxidative damage, regulating osmolytes concentration, and delaying leaf senescence [98, 99], in addition to alterations in the cell wall due to indirect effects of auxin [100]. In light of previous findings, NO application as a priming agent has been found to induce plants tolerance against salinity stress through the modulation of interconnected stress-responsive pathways [101].

Among the most common injuries in plants induced by environmental stresses is ROS hyper-accumulation. Our data showed an enhancement in MDA contents in shoot under salinity stress. The membrane lipids are among the most cellular components oxidized and degraded by ROS, so, concentrations of MDA can increase, indicating injury in the plant cell membranous system [102]. This MDA increment could also induce antioxidants to come off to neutralize ROS production ensuing from salt stress.

Exogenous individually or successively, application of SNP and CaCl_2_ as priming agents retarded the production of MDA within the wheat shoot system exposed to salinity. These findings were in accordance with many studies that reported the reduction in H_2_O_2_ and MDA by SNP treatments throughout salt stress [87, 103, 104].

Under salt stress’s deleterious impacts, plant undergoes osmotic regulation through increased potential osmolyte synthesis, such as proline in the cytosol and organelles. Shoot system proline, a very important secondary metabolite, performs dual functions in plants as an osmo-protectant in addition to being an antioxidant [105]. Our data showed that Proline contents decreased in shoot under salinity stress. It is previously reported that under salinity stress, a high proline concentration acts as a substitute for water to stabilize and protect the cellular structures through their hydrogen bonding as well as hydrophobic interactions, which prevent the dehydration of membranes [106].

The accumulation of proline seems to be a strong defensive strategy against osmotic stress. It regulates the pH of the cytosol and scavenges free radicals by acting as a non-enzymatic antioxidant as well as an active osmolyte [107]. Increased accumulation of proline due to priming treatments might have boosted the antioxidative mechanisms by acting as a direct ROS scavenger or by playing an effective role as a signaling molecule [108]. In the present investigation, an increasing trend in cellular proline contents was observed when seed priming with SNP and CaCl_2_, particularly successive priming with CaCl_2_ + SNP, which activated a higher protection for plants under environmental stresses. SNP and CaCl_2_ could trigger the accumulation of proline, which may induce wheat plant salinity tolerance through the adjustment of osmotic stress by maintaining a greater cellular water content that cause better growth in wheat plants.

successive priming with CaCl_2_ + SNP and SNP + CaCl_2_ enhanced the content of phenolics in the shoot. Phenolic substances, through their ability to scavenge free radicals, may serve as potent antioxidants in addition to substrates for many antioxidant enzymes as well [109]. Under osmotic stress, plant possess a wide range of non-enzymatic antioxidants to quench ROS [110]. From the current data, the positive impact of successive priming could be observed.

Also, SNP + CaCl_2_ and CaCl_2_ + SNP successive priming achieved the highest value of total antioxidants contents in shoot under salinity stress, and this is another important indicator for the potential positive effect of the successive priming technique in enhancing antioxidant activity in plants under salinity stress. The results showed that applying successive priming agents stimulated the production of total antioxidants content in the leaves of wheat plants in relation to corresponding stressed treatments. These findings are in high accordance with our aforementioned results of phenolics, proline, and MDA. An enhancement of antioxidant capacity by the application of priming agents might protect plants under salinity stress conditions [111, 112].

The priming agents increased the activity of APX and CAT enzymes in the leaves. It can be concluded that priming agents, particularly successive priming, could lower ROS generation on wheat plants under salinity stress through increasing APX and CAT activity, thus protecting against oxidative damage. CAT activity has been reported to be negatively correlated with the degree of damage to plasmalemma, chloroplast, and mitochondrial membrane systems and positively related to the indices of stress resistance [113]. According to Jaleel et al. [114] CaCl_2_-treated seedlings maintain higher levels of CAT activities and lower levels of lipid peroxidation and POX activity [114]. A previous study reported that plant pretreatment with SNP could increase antioxidant enzyme activity of POD, CAT, and APX in plant leaves and root [115]. Moreover, it was reported that the main role of NO is to enhance the antioxidation defense system of plants by inducing the antioxidant enzyme activities of CAT, SOD, POD, APX, as well as glutathione reductase [116]. These findings are in accordance with those of Mohsenzadeh and Zohrabi [117], who reported the induction of antioxidative enzymes SOD, POD, CAT and APX as a consequence of SNP application. It was assumed that the SNP role could be achieved by improving the capability of scavenging free radicals and mitigating oxidation damage, along with lower MDA contents. Jabeen et al. [118] stated that under salinity stress, the application of SNP increased the activities of SOD, CAT, POD and APX. External application of SNP may help plants withstand salt stress through stimulating gene expression associated with antioxidant enzymes [119].

Priming treatments in this study increased the shoot contents of soluble proteins under salinity stress. The accumulation of proteins in plants under salt stress conditions may support a re-utilized form of stored nitrogen that can be used later to play a crucial role in osmotic adjustment. Proteins may be saved constitutively at low concentrations or may be synthesized de novo as a consequence of salinity stress. Hasegawa et al. [120] concluded that a number of proteins induced by salinity are cytoplasmic, which in turn can cause alterations in the viscosity of the cell cytoplasm. Habib et al. [68] reported a similar increasing trend in total cellular content of soluble protein in two wheat cultivars as a result of external application SNP or SNP + H_2_O_2_ as seed priming agents, which improved a greater protection under stressed conditions.

## Conclusion

Pretreatment of wheat grains with SNP + CaCl_2_ as successive priming treatment has shown potential in enhancing the tolerance of wheat plants to salinity stress by suppressing the burst of ROS. The control of oxidative stress is evident from the increased levels of phenolics, enzymatic antioxidants (CAT & APX), and total antioxidants. The successive priming with SNP + CaCl_2_ has been found to improve water relations (CWP & IWP), increase potassium content in shoot dry weight, and consequently enhance plant productivity and yield quality, including seed index, grain yield, and grain nitrogen content. These findings fulfilled the study’s aims, as the results answered the aforementioned questions. Our data revealed that successive priming improved water relations (Eta, IWA, CWP& IWP) and wheat plant growth and productivity under salinity stress more than individual priming treatments. Successive priming enhanced stress memory of salt tolerance in wheat, relatively, when compared to unprimed state. However, different seed priming techniques still need to be investigated for precise and reliable applications of this approach.

## Data Availability

The datasets used and/or analysed during the current study are available from the corresponding author on reasonable request.

## Abbreviations

NO: Nitric oxide
SNP: Sodium nitroprusside
ETa: Water consumptive use
IWA: Irrigation water applied
IWP: Irrigation water productivity

## References

[1] Astapati AD. Nath S The complex interplay between plant-microbe and virus interactions in sustainable agriculture: Harnessing phytomicrobiomes for enhanced soil health, designer plants, resource use efficiency, and food security. Crop Design. 2023;12(12):100028. 10.3390/plants12122307. 10.3390/plants12122307

[2] Teh SY, Koh HL. Climate change and soil salinization: impact on agriculture, water and food security. IJAFP. 2016;2:1–9.

[3] Gupta B, Huang B. Mechanism of salinity tolerance in plants: physiological, biochemical, and molecular characterization. Int J Genomics. 2014;701596:1–18. 10.1155/2014/701596 PMC3996477 24804192

[4] IPCC. Climate change 2014: synthesis report. In: Pachauri RK, Meyer LA, editors. Contribution of Working Groups I. IPCC, Geneva Switzerland p: II and III to the Fifth Assessment Report of the Intergovernmental Panel on Climate Change; 2014. p. 151r.

[5] Laifa I, Hajji M, Farhat N, Elkhouni A, Smaoui A, M’nif A, Hamzaoui AH, Savoure A, Abdelly C, Zorrig W,. Beneficial effects of silicon (Si) on sea barley (*Hordeum marinum*Huds.) under salt stress. SILICON. 2021;13:4501–17. 10.1007/s12633-020-00770-1

[6] Bohnert HJ, Nelson DE, Jensen RG. Adaptations to environmental stresses. Plant Cell. 1995;7(7):1099–111. 10.1105/tpc.7.7.1099. 12242400 10.1105/tpc.7.7.1099 PMC160917

[7] Ellouzi H, Rabhi M, Khedher S, Debez A, Abdelly C, Zorrig W. Silicon seed priming enhances salt tolerance of barley seedlings through early ROS detoxification and stimulation of antioxidant defence. SILICON. 2023;15(1):37–60. 10.1007/s12633-022-02001-1. 10.1007/s12633-022-02001-1

[8] Ding Z, Kheir AM, Ali OA, Hafez EM, ElShamey EA, Zhou Z, Wang B, Lin X, Ge Y, Fahmy AE, Seleiman MF. A vermicompost and deep tillage system to improve saline-sodic soil quality and wheat productivity. J Environ Manage. 2020;277: 111388. 10.1016/j.jenvman.2020.111388. 33002812 10.1016/j.jenvman.2020.111388

[9] Yi J, Li H, Zhao Y, Shao M, Zhang H,... Liu M,. Assessing soil water balance to optimize irrigation schedules of flood-irrigated maize fields with different cultivation histories in the arid region. Agric Water Manag. 2022;265: 107543. 10.1016/j.agwat.2022.107543. 10.1016/j.agwat.2022.107543

[10] Hu Q, Zhao Y, Hu X, Qi J, Suo L, Pan Y,... Chen X,. Effect of saline land reclamation by constructing the “Raised Field -Shallow Trench” pattern on agroecosystems in Yellow River Delta. Agric Water Manag. 2022;261: 107345. 10.1016/j.agwat.2021.107345. 10.1016/j.agwat.2021.107345

[11] Kranner I, Minibayeva FV, Beckett RP, Seal CE. What is stress? Concepts, definitions and applications in seed science. New Phytol. 2010;188(3):655–73. 10.1111/j.1469-8137.2010.03461.xr. 20854396 10.1111/j.1469-8137.2010.03461.xr

[12] Wojtyla L, Lechowska K, Kubala S, Garnczarska M. Molecular processes induced in primed seeds—increasing the potential to stabilize crop yields under drought conditions. J Plant Physiol. 2016;203:116–26. 10.1016/j.jplph.2016.04.008. 27174076 10.1016/j.jplph.2016.04.008

[13] Jacques C, Salon C, Barnard RL, VernoudV PM. Drought stress memory at the plant cycle level: A review. Plants. 2021;10(9):1873. 10.3390/plants10091873. 34579406 10.3390/plants10091873 PMC8466371

[14] Ahmad P, Ahanger MA, Alyemeni MN, Wijaya L, Alam P, Ashraf M. Mitigation of sodium chloride toxicity in Solanum lycopersicum L. by supplementation of jasmonic acid and nitric oxide. J Plant Interact. 2018;13(1):64–72. 10.1080/17429145.2018.1424272. 10.1080/17429145.2018.1424272

[15] Bruce TJ, Matthes MC, Napier JA, Pickett JA. Stressful “memories” of plants: evidence and possible mechanisms. Plant Sci. 2007;173(6):603–8. 10.1016/j.plantsci.2007.09.002. 10.1016/j.plantsci.2007.09.002

[16] Martinez-Medina A, Flors V, Heil M, Mauch-Mani B, Pieterse CM, Pozo MJ, Conrath U. Recognizing plant defense priming. Trends plant sci. 2016;21(10):818–22. 10.1016/j.tplants.2016.07.009. 27507609 10.1016/j.tplants.2016.07.009

[17] Xia H, Liu X, Wang Y, Lin Z, Deng H, Wang J, Lin L, Deng Q, Lv X, Xu K, Liang D. 24-Epibrassinolide and nitric oxide combined to improve the drought tolerance in kiwifruit seedlings by proline pathway and nitrogen metabolism. Sci Hortic. 2022;297: 110929. 10.1016/j.scienta.2021.110929. 10.1016/j.scienta.2021.110929

[18] Lutts S, Benincasa P, Wojtyla L, Kubala S, Pace R, Lechowska K, Quinet M, Garnczarska M. Seed priming: new comprehensive approaches for an old empirical technique. New challenges in seed biology-basic and translational research driving seed technology. 2016;46(10.5772):4420.

[19] Singh HRK, JassalJ Kang S, Sandhu H, Kang Grewal K. Seed priming techniques in field crops-A review. Agric Rev. 2015;36(4):251–64.

[20] Jisha K, Vijayakumari K, Puthur JT. Seed priming for abiotic stress tolerance: an overview. Acta Physiol Plant. 2013;35(5):1381–96. 10.1007/s11738-012-1134-6. 10.1007/s11738-012-1134-6

[21] Sivanandhan G, Selvaraj N, Ganapathi A, Manickavasagam M. Effect of nitrogen and carbon sources on in vitro shoot multiplication, root induction and withanolides content in Withania somnifera (L.) Dunal. Acta Physiol Plant. 2015;37(2):1–10. 10.1007/s11738-014-1758-7

[22] Basit F, Ulhassan Z, Mou Q, Nazir MM, Hu J, Hu W, Song W, Sheteiwy MS, Zhou W, Bhat JA, Jeddi K. Seed priming with nitric oxide and/or spermine mitigate the chromium toxicity in rice (*Oryza sativa*) seedlings by improving the carbon-assimilation and minimising the oxidative damages. Funct Plant Biol. 2022;50(2):121–35. 10.1071/FP21082. 10.1071/FP21082 35057906

[23] Hayat S, Hasan SA, Mori M, Fariduddin Q, Ahmad A. Nitric oxide: chemistry, biosynthesis, and physiological role. Nitric oxide plant physiol. 2010;69(14):1–15. 10.1016/j.niox.2009.10.009. 10.1016/j.niox.2009.10.009

[24] Habib NOMAN, Ashraf MUHAMMAD, Ahmad MSA. Enhancement in seed germinability of rice (Oryza sativa L.) by pre-sowing seed treatment with nitric oxide (NO) under salt stress. Pak J Bot. 2010;42(6):4071–8. 10.1016/j.jplph.2016.04.008. 10.1016/j.jplph.2016.04.008

[25] Begara-Morales JC, Sánchez-Calvo B, Chaki M, Mata-Pérez C, Valderrama R, Padilla MN, Barroso JB. Differential molecular response of monodehydroascorbate reductase and glutathione reductase by nitration and S-nitrosylation. J Exp Bot. 2015;66(19):5983–96. 10.1093/jxb/erv301. 26116026 10.1093/jxb/erv301 PMC4566986

[26] Manai J, Gouia H, Corpas FJ. Redox and nitric oxide homeostasis are affected in tomato (*Solanum lycopersicum*) roots under salinity-induced oxidative stress. J Plant Physiol. 2014;171(12):1028–35. 10.1016/j.jplph.2014.03.014. 24974329 10.1016/j.jplph.2014.03.014

[27] Habib N, Ashraf M, Shahbaz M. Effect of exogenously applied nitric oxide on some key physiological attributes of rice (*Oryza sativa L*.) plants under salt stress. Pak J Bot. 2013;45:1563–9. 10.1016/j.jplph.2016.04.008. 10.1016/j.jplph.2016.04.008

[28] Zheng C, Jiang D, Liu F, Dai T, Liu W, Jing Q, Cao W. Exogenous nitric oxide improves seed germination in wheat against mitochondrial oxidative damage induced by high salinity. Environ Exp Bot. 2009;67(1):222–7. 10.1016/j.envexpbot.2009.06.005. 10.1016/j.envexpbot.2009.06.005

[29] Adnan M, Shah Z, Fahad S, Arif M, Alam M, Khan IA, Mian IA, Basir A, Ullah H, Arshad M, Rahman IU, Saud S, Ihsan MZ, Jamal Y, Amanullah HM, Nasim Hammad W. Phosphate-solubilizing bacteria nullify the antagonistic effect of soil calcification on bioavailability of phosphorus in alkaline soils. Sci Rep. 2017;7(1):16131. 10.1038/s41598-017-16392-7. 29170494 10.1038/s41598-017-16392-7 PMC5701022

[30] Wang J, Song J, Wu XB, Deng QQ, Zhu ZY, Ren MJ, Ye M, Zeng RS. Seed priming with calcium chloride enhances wheat resistance against wheat aphid *Schizaphis graminum* Rondani. Pest Manag Sci. 2021;77(10):4709–18. 10.1002/ps.6743. 34146457 10.1002/ps.6743

[31] Hussain M, Farooq M, Sattar A, Ijaz M, Sher A, Ul-Allah S. Mitigating the adverse effects of drought stress through seed priming and seed quality on wheat (*Triticum aestivum L.*) productivity. Pak J Agr Sci. 2018;55:313–9.

[32] Wang Y, Shen C, Jiang Q, Wang Z, Gao C, Wang W. Seed priming with calcium chloride enhances stress tolerance in rice seedlings. Plant Sci. 2022;323: 111381. 10.1016/j.plantsci.2022.111381. 35853520 10.1016/j.plantsci.2022.111381

[33] Zhang T, Song B, Han G, Zhao H, Hu Q, Zhao, Y.,... Liu, H. Effects of coastal wetland reclamation on soil organic carbon, total nitrogen, and total phosphorus in China: A meta-analysis. Land Degrad Dev. 2023;34(11):3340–9. 10.1002/ldr.4687. 10.1002/ldr.4687

[34] Qiu S, Yang H, Zhang S, Huang S, Zhao S, Xu, X.,... Banwart, S. A. Carbon storage in an arable soil combining field measurements, aggregate turnover modeling and climate scenarios. CATENA. 2023;220: 106708. 10.1016/j.catena.2022.106708. 10.1016/j.catena.2022.106708

[35] Du K, Huang J, Wang W, Zeng Y, Li X, Zhao F. Monitoring Low-Temperature Stress in Winter Wheat Using TROPOMI Solar-Induced Chlorophyll Fluorescence. IEEE Trans Geosci Remote Sens. 2024;62:1–11. 10.1109/TGRS.2024.3351141. 10.1109/TGRS.2024.3351141

[36] Yang T, Zhang Y, Guo L, Li D, Liu A, Bilal M, Wang P. Antifreeze Polysaccharides from Wheat Bran: The Structural Characterization and Antifreeze Mechanism. Biomacromolecules. 2024. 10.1021/acs.biomac.3c00958. 38388358 10.1021/acs.biomac.3c00958

[37] Ali Q, Daud MK, Haider MZ, Ali S, Rizwan M, Aslam N, Zhu SJ. Seed priming by sodium nitroprusside improves salt tolerance in wheat (*Triticum aestivum L*.) by enhancing physiological and biochemical parameters. Plant Physiol Biochem. 2017;119:50–8. 10.1016/j.plaphy.2017.08.010r. 28843888 10.1016/j.plaphy.2017.08.010r

[38] Gui YW, Sheteiwy MS, Zhu SG, Batool A, Xiong YC. Differentiate effects of non-hydraulic and hydraulic root signaling on yield and water use efficiency in diploid and tetraploid wheat under drought stress. Environ Exp Bot. 2020;181(4): 104287.

[39] Shiferaw B, Smale M, Braun HJ, Duveiller E, Reynolds M, Muricho G. Crops that feed the world 10 past successes and future challenges to the role played by wheat in global food security. Food Secur. 2013;5:291–317. 10.1007/s12571-013-0263-y

[40] Kiss I. Significance of wheat production in the world economy and position of Hungary in it ABSTRACT: Applied Studies in Agribusiness and Commerce. 2011;5(1–2):115–20.

[41] Alexandratos N, Bruinsma J. World agriculture towards 2030/2050: the 2012 revision. 2012.

[42] Pequeno DN, Hernandez-Ochoa IM, Reynolds M, Sonder K, MoleroMilan A, Robertson RD, Asseng S. Climate impact and adaptation to heat and drought stress of regional and global wheat production. Environ Res Lett. 2021;16(5):054070. 10.1088/1748-9326/abf3f2. 10.1088/1748-9326/abf3f2

[43] Asseng S, Ewert F, Martre P, Rötter RP, Lobell DB, Cammarano D, Kimball BA, Ottman MJ, Wall GW, White JW, Reynolds MP, Alderman PD, Prasad PVV, Aggarwal PK, Anothai J, Basso B, Biernath C, Challinor AJ, De Sanctis G, Doltra J, Fereres E, Garcia-Vila M, Gayler S, Hoogenboom G, Hunt LA, Izaurralde RC, Jabloun M, Jones CD, Kersebaum KC, Koehler A-K, Müller C, Naresh Kumar S, O’Leary Nendel C, G, Olesen JE, Palosuo T, Priesack E, Eyshi Rezaei E, Ruane AC, Semenov MA, Shcherbak I, Stöckle C, Stratonovitch P, Streck T, Supit I, Tao F, Thorburn PJ, Waha K, Wang E, Wallach D, Wolf J, Zhao Z, Zhu Y,. Rising temperatures reduce global wheat production. Nat Clim Change. 2015;5(2):143–7. 10.1038/nclimate2470. 10.1038/nclimate2470

[44] Klute A. Methods of soil analysis. Part 1: Physical and mineralogical methods (2nd edition). American Society of Agronomy Inc., Madison, Wisconsin, USA. 1986. p. 463–78.

[45] Page AI, Miller RH, Keeny DR. Methods of Soil Analysis. Part II. Chemical and Microbiological Methods, 2nd ed. American Society of Agronomy, Madison, WI, USA. 1982. p. 225–46.

[46] Israelsen OW, Hansen VE. Irrigation Principles and Practices, 3^rd^ Edition, John Wiley and Sons Inc", New York, U.S.A. 1962.

[47] Du YD, Cao HX, Liu SQ, Gu XB, Cao YX. Response of yield, quality, water and nitrogen use efficiency of tomato to different levels of water and nitrogen under drip irrigation in Northwestern China. J Integr Agric. 2017;16(5):1153–61. 10.1016/S2095-3119(16)61371-0

[48] Zwart SJ, Bastianssen WGM. Review of measured crop water productivity values for irrigated wheat, rice, cotton and maize. Agric Water Manag. 2014;69(2):115–33. 10.1016/j.agwat.2004.04.007

[49] Lichthenthale H. Chlorophylls end carotenoids: Pigments of photosynthetic bio membranes: Methods Enzymol. San Diego: Academic Press; 1987. p. 350–82.

[50] Padmaja M, Sravanthi M, Hemalatha KPJ. Evaluation of antioxidant activity of two Indian medicinal plants. J Phytol. 2011;3:3.

[51] Hodges DM, DeLong JM, Forney CF, Prange RK. Improving the thiobarbituric acid-reactive-substances assay for estimating lipid peroxidation in plant tissues containing anthocyanin and other interfering compounds. Planta. 1999;207:604–11. 10.1007/s004250050524. 10.1007/s004250050524 28456836

[52] Bates LS, Waldren RP, Teare ID. Rapid determination of free proline for water-stress studies. Plant Soil. 1973;39:205–7. 10.1007/BF00018060

[53] Blois MS. Antioxidant determinations by the use of a stable free radical. Nature. 1958;181:1199. 10.1038/1811199a0

[54] Kofalvi S, Nassuth A. Influence of wheat streak mosaic virus infection on phenylpropanoid metabolism and the accumulation of phenolics and lignin in wheat. Physiol Mol Plant Pathol. 1995;47:365. 10.1006/pmpp.1995.1065

[55] Aebi H. Catalase *in vitro*: Methods Enzymol. Academic Press. 1984. p. 121–6. 10.1016/s0076-6879(84)05016-3 6727660

[56] Tatiana Z, Yamashita K, Matsumoto H. Iron deficiency induced changes in ascorbate content and enzyme activities related to ascorbate metabolism in cucumber roots. Plant Cell Physiol. 1999;40:273–80. 10.1093/oxfordjournals.pcp.a029538

[57] Lowry OH, Rosebrough NJ, Farr AL, Randall RJ. Protein measurement with Folin phenol reagent. J Biol Chem. 1951;193:265. 14907713 10.1016/S0021-9258(19)52451-6

[58] Allen SE, Grimshaw HM, Parkinson JA, Quarmby C. Chemical analysis of ecological materials. Blackwell Scientific Publications. 1974.

[59] Williams V, Twine S. Flame photometeric method for element analysis. Modern methods of plant analysis. 1960;5:3–5.

[60] Alves RC, Nicolau CM, Checchio MV, Junior GSS, Oliveira FA, Prado RM, Grata PL. Salt stress alleviation by seed priming with silicon in lettuce seedlings: an approach based on enhancing antioxidant responses. Bragantia. 2019;79:19–29. 10.1590/1678-4499.20190360

[61] Yan K, Shao H, Shao C, Chen P, Zhao S, Brestic M, Chen X. Physiological adaptive mechanisms of plants grown in saline soil and implications for sustainable saline agriculture in coastal zone. Acta Physiol Plant. 2013;35:2867–78. 10.1007/s11738-013-1325-7

[62] Semiz G, Suarez D, Ünlükara A, Yurtseven E. Interactive effects of salinity and N on pepper (Capsicum annuum L.) yield, water use efficiency and root zone and drainage salinity. J Plant Nutr. 2014;37:595–610. 10.1080/01904167.2013.867985

[63] Zhang WW, Meng JJ, Xing JY, Yang S, Guo F, Li XG, Wan SB. The K^+^/H^+^ antiporter *AhNHX1* improved tobacco tolerance to NaCl stress by enhancing K^+^ retention. J Plant Biol. 2017;60:259–67. 10.1007/s12374-016-0905-7

[64] Dong G, Guo J, Chen J, Sun G, Gao S, Hu L, Wang Y. Effects of spring drought on carbon sequestration, evapotranspiration and water use efficiency in the Songnen meadow steppe in north-east China. Ecohydrology. 2011;4(2):211–24. 10.1002/eco.200

[65] Harsharn SG. Water uptake, water use efficiency, plant growth and ionic balance of wheat, barley, canola and chickpea plants on asodic vertosol with variable subsoil Na Cl salinity. Agric Water Manag. 2010;97(1):148–56. 10.1016/j.agwat.2009.09.002. 10.1016/j.agwat.2009.09.002

[66] Tcherkez G, Mahé HM. ^12^C/^13^C fraction at ions in plant primary metabolism. Trends Plant Sci. 2011;16(9):499–506. 10.1016/j.tplants.2011.05.006. 21705262 10.1016/j.tplants.2011.05.006

[67] Malko MM, Khanzada A, Wang X, Samo A, Li Q, Jiang D, Cai J. Chemical treatment refines drought tolerance in wheat and its implications in changing climate: A review. Plant Stress. 2022;6: 100118. 10.1016/j.stress.2022.100118

[68] Habib N, Ali Q, Ali S, Javed MT, Haider MZ, Perveen R, Shahid MR, Rizwan M, Abdel-Daim MM, Elkelish A, Bin-Jumah M. Use of Nitric Oxide and Hydrogen Peroxide for Better Yield of Wheat (Triticum aestivum L) under Water Deficit Conditions: Growth, Osmoregulation, and Antioxidative Defense Mechanism. Plants. 2020;9:285. 10.3390/plants9030285. 32098385 10.3390/plants9030285 PMC7076392

[69] Khushboo BK, Singh P, Raina M, Sharma V, Kumar D. Exogenous application of calcium chloride in wheat genotypes alleviates negative effect of drought stress by modulating antioxidant machinery and enhanced osmolyte accumulation. In Vitro Cell Dev-Pl. 2018;54:495–507. 10.1007/s11627-018-9912-3

[70] Tahjib-Ul-Arif M, Roy PR, Sohag AAM, Afrin S, Rady MM, Hossain MA. Exogenous calcium supplementation improves salinity tolerance in BRRI Dhan28; a salt susceptible high-yielding *Oryza sativa* cultivar. J Crop Sci Biotechnol. 2018;21(4):383–94. 10.1007/s12892-018-0194-6. 10.1007/s12892-018-0194-6

[71] Ashraf M, Harris PJC. Abiotic stresses: plant resistance through breeding and molecular approaches. New York: Haworth Press; 2005.

[72] Tuteja N. Mechanisms of high salinity tolerance in plants. Meth Enzymol. 2007;428:419–38. 10.1016/S0076-6879(07)28023-9. 10.1016/S0076-6879(07)28023-9 17875432

[73] Munns R, Tester M. Mechanisms of Salinity Tolerance. Annu Rev Plant Biol. 2008;59:651–81. 10.1146/annurev.arplant.59.032607.092911. 18444910 10.1146/annurev.arplant.59.032607.092911

[74] Basyuni M, Wasilah M, Hasibuan PAZ, Sulistiyono N, Sumardi S, Bimantara Y, Hayati R, Sagami H, Oku H. Salinity and subsequent freshwater influences on the growth, biomass, and polyisoprenoids distribution of Rhizophora apiculata seedlings. Biodiversitas. 2019;20:288–95.

[75] Munns R, Day DA, Fricke W, Watt M, Arsova B, Barkla BJ, Bose J, Byrt CS, Chen ZH, Foster KJ, Gilliham M, Henderson SW, Kronzucker CLD, Jenkins HJ, Miklavcic SJ, Plett D, Roy SJ, Shabala S, Shelden MC, Soole KL, Taylor NL, Tester M, Wege S, Wegner LH, Tyerman SD. Energy costs of salt tolerance in crop plants. New Phytol. 2020;225(3):1072–90. 31004496 10.1111/nph.15864

[76] Hameed A, Farooq T, Hameed A, Sheikh MA. Sodium nitroprusside mediated priming memory invokes water-deficit stress acclimation in wheat plants through physio-biochemical alterations. Plant Physiol Biochem. 2021;160:329–40. 33548800 10.1016/j.plaphy.2021.01.037

[77] Ashraf M, Harris PJC. Potential biochemical indicators of salinity tolerance in plants. Plant Sci. 2004;166:3–16. 10.1016/j.plantsci.2003.10.024

[78] Maqsood T, Akhtar J, Farooq MR, Haq MA, Saqib ZA. Biochemical attributes of salt tolerant and salt sensitive maize cultivars to salinity and potassium nutrition. Pak J Agric Sci. 2008;45(1):1–5.

[79] Tian Y, Guan B, Zhou D, Yu J, Li G, Lou Y. Responses of Seed Germination, Seedling Growth, and Seed Yield Traits to Seed Pretreatment in Maize (Zea mays L.). Sci World J. 2014;2014(1):834630. 10.1155/2014/834630. 10.1155/2014/834630 PMC4100373 25093210

[80] Toklu F, Baloch FS, Karaköy T, Özkan H. Effects of different priming applications on seed germination and some agro-morphological characteristics of bread wheat (*Triticum aestivum L*.)". Turk J Agric For. 2015;39:1005–13. 10.3906/tar-1404-41

[81] Ibrahim EA. Seed priming to alleviate salinity stress in germinating seeds. Plant Physiol. 2016;192:38–46. 10.1016/j.plaphy.2016.01.004. 10.1016/j.plaphy.2016.01.004 26812088

[82] Garg N, Bharti A. Salicylic acid improves arbuscular mycorrhizal symbiosis, and chickpea growth and yield by modulating carbohydrate metabolism under salt stress. Mycorrhiza. 2018;28:727–46. 10.1007/s00572-018-0853-6. 30043257 10.1007/s00572-018-0853-6

[83] Abdel Latef AA, Chaoxing H. Does the inoculation with Glomus mosseae improves salt tolerance in pepper plants? J Plant Growth Regul. 2014;33:644–53. 10.1007/s00344-014-9416-3. 10.1007/s00344-014-9416-3

[84] Mostofa MG, Fujita M, Tran LSP. Nitric oxide mediates hydrogen peroxide-and salicylic acid-induced salt tolerance in rice (*Oryza sativa* L.) seedlings. Plant Growth Regul. 2015;77:265–77. 10.1007/s10725-015-0069-6. 10.1007/s10725-015-0069-6

[85] Ahmad Z, Tahir S, Abid M, Amanullah M. Salt-induced variations in physiological parameters and nutrient concentrations of two wheat cultivars. Commun Soil Sci Plant Anal. 2014;45:29–41. 10.1080/00103624.2013.855840. 10.1080/00103624.2013.855840

[86] Habib N, Ashraf M. Effect of exogenously applied nitric oxide on water relations and ionic composition of rice (*Oryza sativa* L.) plants under salt stress. Pak J Bot. 2014;46:111–6.

[87] Gupta P, Srivastava S, Shekhar Seth C. 24-epibrassinolide and sodium nitroprusside alleviate the salinity stress in *Brassica juncea L*. cv. Varuna through cross talk among proline, nitrogen metabolism and abscisic acid. Plant Soil. 2017;411:483–98. 10.1007/s11104-016-3023-2. 10.1007/s11104-016-3023-2

[88] Hanafy AAH, Mohamed HFY, Orabi IOA, EL-Hefny AM,. Influence of gamma rays, humic acid and sodium nitroprusside on growth, chemical constituents and fruit quality of snap bean plants under different soil salinity levels. Biosci Res. 2018;15(2):575–88.

[89] Tester M, Davenport R. Na^+^ tolerance and Na^+^ transport in higher plants. Ann Bot. 2003;91:503–27. 10.1093/aob/mcg058r. 12646496 10.1093/aob/mcg058r PMC4242248

[90] Chen J, Xiao Q, Wu F, Dong X, He J, Pei Z, Zheng H. Nitric oxide enhances salt secretion and Na^+^ sequestration in a mangrove plant *Avicennia marina* through increasing the expression of H^+^-ATPase and Na^+^/H^+^ antiporter under high salinity. Tree physiol. 2010;30:1570–85. 21030403 10.1093/treephys/tpq086

[91] Shaki F, Maboud HE, Niknam V. Effects of salicylic acid on hormonal cross talk, fatty acids profile, and ions homeostasis from salt-stressed safflower. J Plant Interact. 2019;14(1):340–6. 10.1080/17429145.2019.1635660

[92] Ahanger MA, Aziz U, Alsahli AA, Alyemeni MN, Ahmad P. Influence of exogenous salicylic acid and nitric oxide on growth, photosynthesis, and ascorbate-glutathione cycle in salt stressed Vigna angularis. Biomolecules. 2020;10(1):42. 10.3390/biom10010042 PMC7022326 31888108

[93] Miranda RS, Gomes-Filho E, Prisco JT, Alvarez-Pizarro JC. Ammonium improves tolerance to salinity stress in Sorghum bicolor plants. Plant Growth Regul. 2016;78:121–31. 10.1007/s10725-015-0079-1

[94] Zhang X, Pei D, Chen S, Sun H, Yang Y. Performance of double-cropped winter wheat-summer maize under minimum irrigation in the North China Plain. Agron J. 2006;98(6):1620–6. 10.2134/agronj2005.0358

[95] Shirazi MU, Ashraf MY, Khan MA, Naqvi MH. Potassium induced salinity tolerance in wheat. Int J Environ Sci Technol. 2005;2:233–6. 10.1007/BF03325881

[96] Abdul Majid S, Asghar R, Murtaza G. Potassium- calcium interrelationship linked to drought tolerance in wheat (Triticum aestivum L.). Pak J Bot. 2007;39(5):1609–31.

[97] Mahmood A. A new rapid and simple method of screening wheat plants at early stage of growth for salinity tolerance. Pak J Bot. 2009;41(1):255–62. 10.1007/s12633-021-00807-2. 10.1007/s12633-021-00807-2

[98] Lin Y, Liu Z, Shi Q, Wang X, Wei M, Yang F. Exogenous nitric oxide (NO)increased antioxidant capacity of cucumber hypocotyl and radicle under salts tress. Sci Hortic (Amsterdam). 2012;142:118–27. 10.1016/j.scienta.2012.04.032. 10.1016/j.scienta.2012.04.032

[99] Ahmad P, Abdel Latef AA, Hashem A, Abd-Allah EF, Gucel S, Tran LSP. Nitric oxide mitigates salt stress by regulating levels of osmolytes and antioxidant enzymes in chickpea. Front Plant Sci. 2016;7:1–11. 10.3389/fpls.2016.00347. 27066020 10.3389/fpls.2016.00347 PMC4814448

[100] Bõhm FMLZ, de Ferrarese MLL, Zanardo DIL, Magalhaes JR, Ferrarese-Filho O. Nitric oxide affecting root growth, lignification and related enzymes in soybean seedlings. Acta Physiol Plant. 2010;32:1039–46. 10.1007/s11738-010-0494-x

[101] Hossain MA, Burritt DJ, Fujita M. Cross-stress tolerance in plants: molecular mechanisms and possible involvement of reactive oxygen species and methylglyoxal detoxification systems. In: Tuteja N, Gill SS, editors. Abiotic stress response in plants. New York: Wiley; 2016. p. 323–75.

[102] Kaya C, Akram A, Ashraf M, Sonmez O. Exogenous application of humic acid mitigates salinity stress in maize (Zea mays L.) plants by improving some key physio-biochemical attributes. Cereal Res Commun. 2018;46(1):67–78r. 10.1556/0806.45.2017.064

[103] Akram N, Iqbal M, Muhammad A, Ashraf M, Al-Qurainy F, Shafiq S. Aminolevulinic acid and nitric oxide regulate oxidative defense and secondary metabolisms in canola (*Brassica napus*L.) under drought stress. Protoplasma. 2018;225:163–74. 10.1007/s00709-017-1140-x 28699026

[104] Hasanuzzaman M, Nahar K, Rahman A, Inafuku M, Oku H, Fujits M. Exogenous nitric oxide donor and arginine provide protection against short-term drought stress in wheat seedlings. Physiol Mol Biol Plants. 2018;24(6):993–1004. 30425418 10.1007/s12298-018-0531-6 PMC6214438

[105] Habib N, Akram MS, Javed MT, Azeem M, Ali Q, Shaheen HL, Ashraf M. Nitric oxide regulated improvement in growth and yield of rice plants grown under salinity: Antioxidant defense system. Appl Ecol Environ Res. 2016;14:91–105. 10.15666/aeer/1405_091105

[106] KaviKishor PB, Sangam S, Amrutha RN, Laxmi PS, Naidu KR, Rao KRSS, Rao S, Reddy KJ, Theriappan P, Sreenivasulu N. Regulation of proline biosynthesis, degradation, uptake and transport in higher plants: Its implications in plant growth and abiotic stress tolerance. Curr Sci. 2005;88:424–38.

[107] Reddy PP. Bio-priming of seeds. Recent Advances in Crop Protection. India: Springer; 2013. p. 83–90.

[108] Hossain MA, Hoque MA, Burritt DJ, Fujita M. Proline Protects Plants Against Abiotic Oxidative Stress: Biochemical and Molecular Mechanisms. In: Oxidative Damage to Plants; Academic Press: Cambridge. USA: MA; 2014. p. 477–522.

[109] Blokhina O, Violainen E, Agerstedt KV. Antioxidants, oxidative damage and oxygen deprivation stress: A review. Ann Bot. 2003;91:179–94. 12509339 10.1093/aob/mcf118 PMC4244988

[110] Ghadakchiasl A, Mozafari AA, Ghaderi N. Mitigation by sodium nitroprusside of the effects of salinity on the morpho-physiological and biochemical characteristics of *Rubusidaeus* under in vitro conditions. Physiol Mol Biol Plants. 2017;23(1):73–83. 28250585 10.1007/s12298-016-0396-5 PMC5313400

[111] Cervilla LM, Blasco B, Rios JJ, Romero L, Ruiz JM. Oxidative stress and antioxidants in tomato (*Solanum lycopersicum*) plants subjected to boron toxicity. Ann Bot. 2007;100:747–56. 17660516 10.1093/aob/mcm156 PMC2749626

[112] Koshiba T, Kobayashi M, Matoh T. Boron nutrition of tobacco BY-2 cells. V. Oxidative damage is the major cause of cell death induced by boron deprivation. Plant Cell Physiol. 2009;50:26–36. 10.1093/pcp/pcn184. 19054807 10.1093/pcp/pcn184 PMC2638710

[113] Qureshi MI, Israr M, Abdin MZ, Iqbal M. Responses of *Artemisia annua* L. to lead and salt induced oxidative stress. Environ Exp Bot. 2005;53:185–93. 10.1016/j.envexpbot.2004.03.014

[114] Jaleel CA, Riadh K, Gopi R, Manivannan P, Inès J, Al-Juburi HJ, ChangXing Z, Hong-Bo S, Panneerselvam R. Antioxidant defense responses: physiological plasticity in higher plants under abiotic constraints. Acta Physiol Plant. 2009;31:427–36. 10.1007/s11738-009-0275-6

[115] Nejadalimoradi H, Nasibi F, Kalantari KM, Zanganeh R. Effect of seed priming with L-arginine and sodium nitroprusside on some physiological parameters and antioxidant enzymes of sunflower plants exposed to salt stress. 2014.

[116] Kaya C, Ashraf M, Sonmez O. Exogenously applied nitric oxide confers tolerance to salinity-induced oxidative stress in two maize (*Zea mays*, L.) cultivars differing in salinity tolerance. Turk J Agric For. 2015;39:909–19. 10.3906/tar-1411-26

[117] Mohsenzadeh S, Zohrabi M. Auxin and sodium nitroprusside effects on wheat antioxidants in salinity. Russ J Plant Physiol. 2018;65:651–7. 10.1134/S1021443718050138

[118] Jabeen Z, Fayyaz HA, Irshad F, Hussain N, Hassan MN, Li J, Alsubeie MS. Sodium nitroprusside application improves morphological and physiological attributes of soybean (Glycine max L) under salinity stress. Plos one. 2021;16(4):e0248207. 33861749 10.1371/journal.pone.0248207 PMC8051766

[119] Ahmad P, Hashem A, Abd-Allah EF, Alqarawi AA, John R, Egamberdieva D, Gucel S. Role of *Trichoderma harzianumin* mitigating NaCl stress in Indian mustard through antioxidative defense system. Front Plant Sci. 2015;6:868. 26528324 10.3389/fpls.2015.00868 PMC4604702

[120] Hasegawa PM, Bressan RA, Zhu JK, Bohnert HJ. Plant cellular and molecular responses to high salinity. Annu. rev. plant physiol. plant mol. Biol. 2000;51:463–99. 10.1146/annurev.arplant.51.1.463 15012199

